# Anticancer Potential of Diruthenium Complexes with Bridging Hydrocarbyl Ligands from Bioactive Alkynols

**DOI:** 10.1021/acs.inorgchem.3c01731

**Published:** 2023-09-15

**Authors:** Giulio Bresciani, Ján Vančo, Tiziana Funaioli, Stefano Zacchini, Tomáš Malina, Guido Pampaloni, Zdeněk Dvořák, Zdeněk Trávníček, Fabio Marchetti

**Affiliations:** † University of Pisa, Dipartimento di Chimica e Chimica Industriale, Via G. Moruzzi 13, I-56124 Pisa, Italy; ‡ Regional Centre of Advanced Technologies and Materials, Czech Advanced Technology and Research Institute, Palacký University, Šlechtitelů 27, CZ-779 00 Olomouc, Czech Republic; § University of Bologna, Dipartimento di Chimica Industriale “Toso Montanari”, Viale del Risorgimento 4, I-40136 Bologna, Italy; ∥ Department of Cell Biology and Genetics, Faculty of Science, Palacký University, Šlechtitelů 27, CZ-779 00 Olomouc, Czech Republic

## Abstract

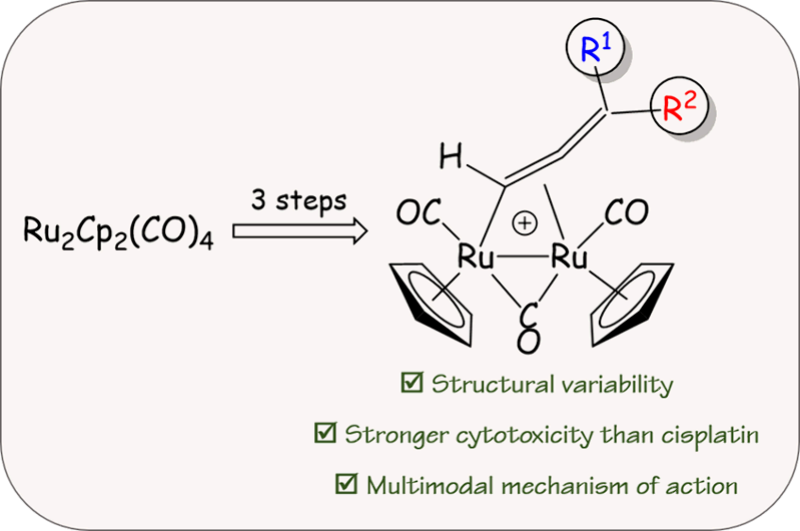

Diruthenacyclopentenone complexes of the general composition [Ru_2_Cp_2_(CO)_2_{μ–η^1^:η^3^-CH=C(C(OH)(R))C(=O)}] (**2a**–**c**; Cp = η^5^-C_5_H_5_) were synthesized in 94–96% yields from the reactions of [Ru_2_Cp_2_(CO)_2_{μ–η^1^:η^3^-C(Ph)=C(Ph)C(=O)}] (**1**) with 1-ethynylcyclopentanol, 17α-ethynylestradiol, and 17-ethynyltestosterone, respectively, in toluene at reflux. Protonation of **2a**–**c** by HBF_4_ afforded the corresponding allenyl derivatives [Ru_2_Cp_2_(CO)_3_{μ–η^1^:η^2^-CH=C=R}]BF_4_ (**3a**–**c**) in 85–93% yields. All products were thoroughly characterized by elemental analysis, mass spectrometry, and IR, UV–vis, and nuclear magnetic resonance spectroscopy. Additionally, **2a** and **3a** were investigated by cyclic voltammetry, and the single-crystal diffraction method was employed to establish the X-ray structures of **2b** and **3a**. The cytotoxicity in vitro of **2b** and **3a**–**c** was evaluated against nine human cancer cell lines (A2780, A2780R, MCF-7, HOS, A549, PANC-1, Caco-2, PC-3, and HeLa), while the selectivity was assessed on normal human lung fibroblast (MRC-5). Overall, complexes exert stronger cytotoxicity than cisplatin, and **3b** (comprising 17α-estradiol derived ligand) emerged as the best-performing complex. Inductively coupled plasma mass spectrometry cellular uptake studies in A2780 cells revealed a higher level of internalization for **3b** and **3c** compared to **2b**, **3a,** and the reference compound RAPTA-C. Experiments conducted on A2780 cells demonstrated a noteworthy impact of **3a** and **3b** on the cell cycle, leading to the majority of the cells being arrested in the G0/G1 phase. Moreover, **3a** moderately induced apoptosis and oxidative stress, while **3b** triggered autophagy and mitochondrial membrane potential depletion.

## Abstract 2

A new class of diruthenium organometallics, including complexes with estradiol fragments, revealed a promising in vitro anticancer activity which appears related to a multimodal mechanism.

## 1 Introduction

**Figure 1:**
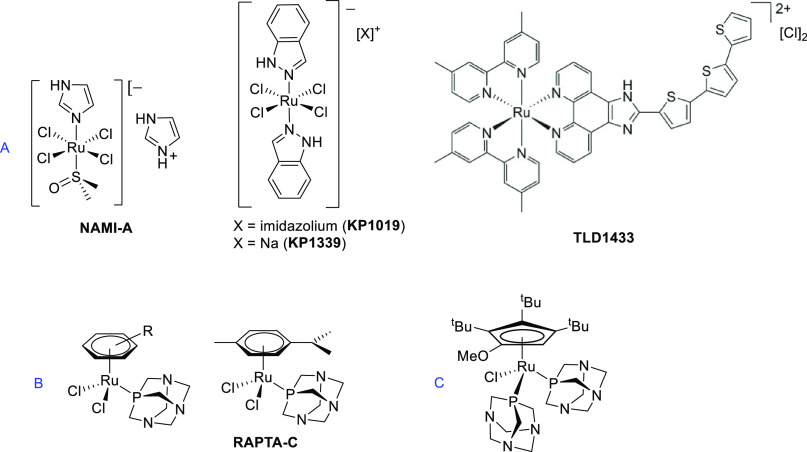
Structures of relevant ruthenium compounds investigated for their anticancer activity. (A) Ru^III^ and Ru^II^ complexes assessed in clinical trials. (B) Generic structure of RAPTA complexes (Ru^II^) and structure of **RAPTA-C**. (C) Cyclopentadienyl bis-PTA complex (Ru^II^).

Extensive research has been dedicated to the exploration of ruthenium compounds as potential candidates for anticancer drugs,^1−3^ and a few ruthenium complexes entered clinical trials for chemotherapy or photodynamic therapy (Figure 1A).^4−6^ Organometallic piano-stool complexes based on the [Ru^II^(η^6^-arene)] core have emerged as possible alternatives to platinum-based chemotherapeutics,^4,7−10^ and in particular complexes belonging to the RAPTA family have shown great promise (Figure 1B).^11−13^ In addition, various ruthenium(II) compounds bearing a η^5^-coordinated cyclopentadienyl ligand (Cp), or its substituted derivatives, have attracted increasing attention in the medicinal field (Figure 1C).^14−19^ Since the Cp ligand is formally anionic, the bond with the ruthenium is strengthened by an electrostatic contribution, supplying robustness to the overall structure. In the pursuit of novel and potent metallodrugs, dinuclear metal complexes could offer significant advantages compared to their corresponding monometallic counterparts.^20,21^ In this regard, several complexes containing two ruthenium(II) arene fragments connected by variable linkers^22−28^ and a diversity of other diruthenium species^29−32^ have been evaluated, showing an interesting activity in several cases. We note that the “diruthenium approach” is not always favorable; for instance, the assembly of two {RuCp(CO)_2_} units with diamine linkers resulted in the absence of cytotoxicity on human cancer cell lines.^33,34^

To the best of our knowledge, diorganoruthenium compounds bearing a direct metal–metal bond have been almost unexplored for their anticancer potential; the commercial [Ru_2_Cp_2_(CO)_4_] serves as a convenient initial compound for synthesizing a wide range of [RuRu] organometallics,^35−38^ for which biological investigations are almost absent in the literature. There are some substantial reasons for encouraging the advance of this piece of chemistry. Indeed, the cooperativity of the (Cp)M–M(Cp) core (M = Ru or Fe) enables the construction, on one bridging site, of functionalized hydrocarbyl ligands otherwise not available on related mononuclear cyclopentadienyl complexes.^35,39−41^ This synthetic strategy offers advantageous opportunities in view of a drug design, such as the facile incorporation within the dimetallic framework of organic fragments playing a specific biological role, and the introduction of a net positive charge enhancing the water solubility of the complex.^35^ Moreover, the interaction with specific biosubstrates may stimulate the dissociation from the ruthenium centers of carbon monoxide molecules, representing a possible adjuvant effect to the anticancer activity, as it has been documented for other metal–carbonyl species.^42−44^

In this work, we present the synthesis, characterization, and evaluation of the anticancer potential of a novel series of diruthenium complexes derived from [Ru_2_Cp_2_(CO)_4_] via the incorporation of alkynes, including two estradiol-containing alkynes. It has to be remarked that tethering a bioactive molecule to an anticancer metal scaffold is a prominent approach to optimize the performance of the complex.^45−49^ This field of research has included the conjugation of metal structures with, inter alia, steroid hormone derivatives, and related examples have appeared in the literature concerning platinum(II),^50,51^ ferrocene,^52^ and ruthenium arene complexes.^53^ Steroidal units may enhance the antiproliferative activity especially against hormone-dependent cancer cells (e.g., breast cancer cells), by facilitating the intracellular drug uptake through steroid receptor systems.^54^

## 2 Results and Discussion

### 2.1 Synthesis and Characterization of Complexes

**Scheme 1:**
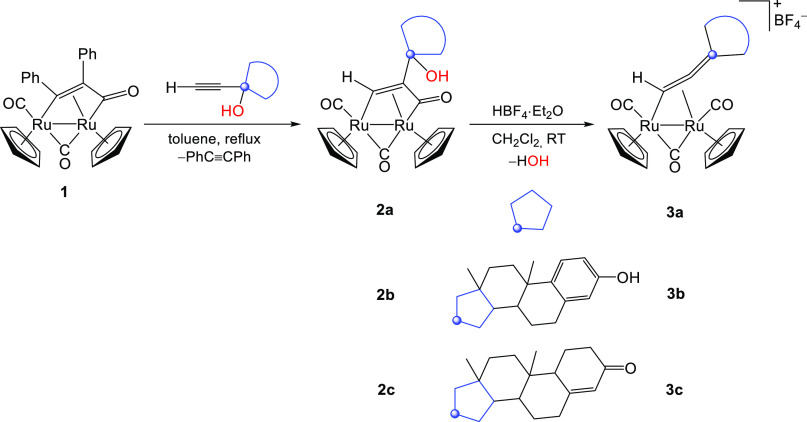
Synthesis of Diruthenacyclopentenone (**2a**–**c**) and Diruthenium μ-Allenyl Complexes from Alkynols with a Cyclic Substituent (**3a**–**c**)

We synthesized the diruthenacyclopentenone complex **1** using a recently published procedure, starting from commercial [Ru_2_Cp_2_(CO)_4_].^55^ It has been established that the {PhCCPh} fragment within **1** is susceptible to thermal exchange with a variety of alkynes.^38,55,56^ Specifically, there is documented evidence that the reactions between **1** and propargyl alcohols of formula HC≡CCRR′OH (RR′ = HH, MeMe, MePh) are regioselective, i.e., the CRR’OH unit in the diruthenacyclopentenone products is placed far from the Cp rings.^57−60^ Then, addition of a strong protonating agent leads to cationic complexes with a bridging allenyl ligand, via H_2_O elimination.^57−59,61^ We exploited this strategy to conjugate 17α-ethynylestradiol and 17-ethynyltestosterone with the {Ru_2_Cp_2_(CO)_3_} scaffold (Scheme 1). Thus, the reactions of **1** with an excess of such two alkynes and 1-ethynylcyclopentanol, which was used as a nonbioactive analog, were carried out in toluene solution under reflux conditions. These reactions resulted in the formation of unprecedented diruthenacyclopentenone species **2a**–**c** in almost quantitative yields. Following this, **2a**–**c** was subjected to reactions with tetrafluoroboric acid in dichloromethane, leading to the corresponding allenyl derivatives **3a**–**c** in high yields.

The IR spectra of **2a**–**c** (dissolved in CH_2_Cl_2_) exhibit a consistent pattern comprising three distinct absorptions, associated with the terminal and bridging carbonyl ligands, as well as the acyl moiety (e.g., for **2b**, at 1976, 1803, and 1751 cm^–1^, respectively). The NMR spectra of **2a**–**c** (in acetone-d_6_, Figures S1 and S6) reveal singular sets of resonances. In the ^1^H NMR spectra, the Cp ligands resonate at ca. 5.5 and 5.2 ppm; these values indicate a cis configuration with respect to the Ru_2_-μ-CO plane, as previously observed for analogous complexes.^37,59^ The hydroxyl group adjacent to the dimetallacycle manifests itself with a broad ^1^H signal around 3.5 ppm. The alkenyl CH moiety is observed at characteristic low chemical shift values [δ(^1^H) = 10.70–10.87 ppm, δ(^13^C) = 151.9–156.0 ppm], thus revealing a bridging alkylidene character.^41,62−66^ In the ^13^C NMR spectrum of **2b**, the CO units have been detected at 237.1 (bridging CO), 219.6 (acyl), and 201.5 ppm (terminal CO), in agreement with literature data on similar complexes.^37,59,67^

**Figure 2:**
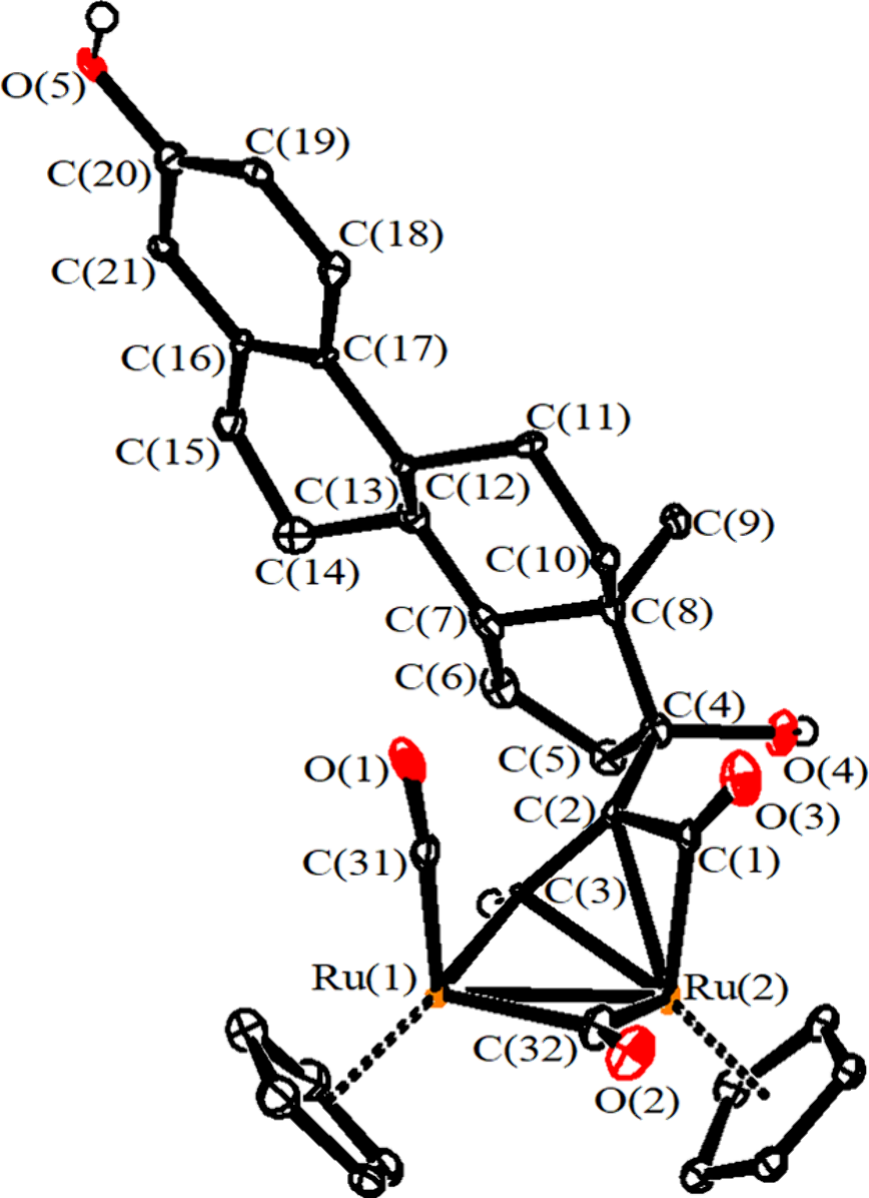
View of the molecular structure of **2b**. Displacement ellipsoids are presented at the 50% probability level. Hydrogen atoms have been excluded for the sake of clarity, with the exception of those attached to oxygen atoms and C(3).

The molecular structure of **2b** was determined through X-ray diffraction analysis (Figure 2, Table 1). Coherently with the NMR data, the structure consists of a cis-Ru_2_Cp_2_(CO)(μ-CO) core bonded to μ–η^1^:η^3^-CH = C(R)C(=O) with R being an estradiol fragment. The presence of the bulky steroid moiety does not significantly affect the geometry of the dimetallacyclopentenone core compared to analogous bimetallic complexes containing less hindered substituents.^37,58,60,68,69^

**Table 1:** Selected Bond Lengths (Å) and Angles (°) for **2b**

Ru(1)–Ru(2)	2.7356(18)	Ru(1)–C(31)	1.854(18)
Ru(1)–C(32)	2.066(18)	Ru(2)–C(32)	2.036(18)
Ru(1)–C(3)	2.054(14)	Ru(2)–C(3)	2.137(14)
Ru(2)–C(1)	1.993(18)	Ru(2)–C(2)	2.208(17)
C(31)–O(1)	1.16(2)	C(32)–O(2)	1.16(2)
C(1)–O(3)	1.18(2)	C(1)–C(2)	1.49(2)
C(2)–C(3)	1.41(2)	C(2)–C(4)	1.51(3)
C(4)–O(4)	1.45(2)	C(20)–O(5)	1.37(2)
Ru(1)–C(32)–Ru(2)	83.7(7)	Ru(1)–C(3)–Ru(2)	81.5(5)
Ru(1)–C(31)–O(1)	174.5(16)	Ru(2)–C(1)–C(2)	77.2(10)
Ru(2)–C(1)–O(3)	144.3(15)	C(2)–C(1)–O(3)	137.9(18)
C(1)–C(2)–C(3)	114.5(15)	Ru(1)–C(3)–C(2)	125.8(12)

The C(3) carbon is positioned almost equidistantly between the two ruthenium atoms [Ru(1)–C(3) 2.054(14) and Ru(2)–C(3) 2.137(14) Å), reflecting its bridging alkylidene nature, in agreement with NMR spectroscopy.

**Scheme 2:**
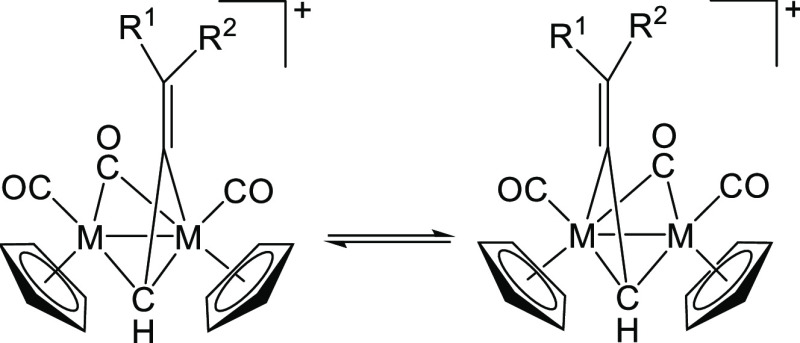
Fluxional Process (σ–π Oscillation) Occurring in Dimetallic μ-Allenyl Complexes in Solution (M = Fe or Ru; R^1^, R^2^ = H, alkyl or Ph)

The IR spectra of **3a**–**c** (dissolved in CH_2_Cl_2_) display three bands corresponding to two terminal and one bridging CO ligands (e.g., for **3a**, at 2039, 2018, and 1871 cm^–1^). On the other hand, the NMR spectra of **3a**–**c** (recorded in acetone-d_6_) at room temperature exhibit broad signals that could not be assigned, implying the occurrence of a fluxional process. There is large evidence in the literature that analogous diiron and diruthenium complexes containing a bridging η^1^:η^2^-allenyl ligand experience rapid oscillation of the allenyl moiety between the two metal atoms (σ–π fluxionality, Scheme 2).^57,58,60^ The same process is common to diiron and diruthenium μ–η^1^:η^2^-alkenyl compounds.^70,71^

**Scheme 3:**
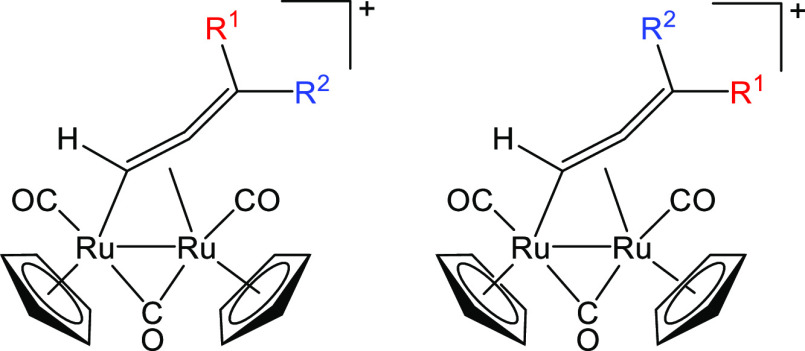
Stereo-Isomerism Observed in Diruthenium μ-Allenyl Complexes (R^1^ ≠ R^2^)

Resolved ^1^H NMR spectra for **3a**–**c** (Figures S7–S9) were recorded at −20 °C, and they unveiled the existence of two distinct resonance sets. The two resonance sets were assigned to cis and trans isomers in **3a** [δ = 6.12, and 5.98 ppm (cis); δ = 5.95 and 5.80 ppm (trans), with the former largely prevailing. Note that analogous μ-allenyl complexes usually display cis configuration of the Cp rings.^57,59,72^ Besides, in diruthenium μ–η^1^:η^2^-alkenyl species, it is common to observe one Cp ^1^H NMR resonance at a higher chemical shift in the trans isomer compared to the cis isomer.^58,73^ According to low-temperature ^1^H NMR data, it can be inferred that **3b** and **3c** exist in solution as a combination of two isomers, both featuring cis geometry of the Cp ligands [e.g., for **3b**: δ = 6.19, 5.99 ppm and 6.18, 6.01 ppm for the two isomers, respectively]. It is presumable that this isomerism arises from the two possible orientations adopted by the cyclic substituent on the allenyl. Consistently, two isomers were previously reported for [Ru_2_(CO)_2_(μ-CO){μ–η^1^:η^2^-CH=C=C(Me)(Ph)}(Cp)_2_]^+^, varying in the orientations of the Me and Ph groups (Scheme 3).^59^

In the ^1^H spectra, the ruthenium-bound CH undergoes a minor shift ongoing from **2a**–**c** to **3a**–**c** (e.g., from 10.87 ppm in **2a** to 10.80 ppm in the *cis* isomer of **3a**), thus indicating that the bridging alkylidene character of such CH carbon is maintained upon dimetallacyclopentenone to allenyl conversion.

**Figure 3:**
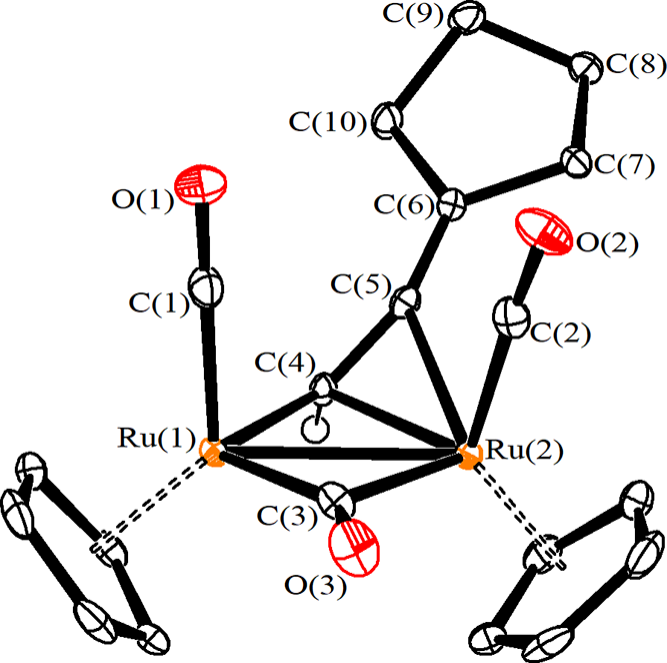
View of the molecular structure of the cation of **3a**. Displacement ellipsoids are presented at the 50% probability level. Hydrogen atoms have been excluded for clarity, with the exception of that attached to C(4).

The structure of **3a** was elucidated by single-crystal X-ray diffraction studies (Figure 3, Table 2). It may be described as a diruthenium complex consisting of a cis-Ru_2_Cp_2_(CO)_2_(μ-CO) core linked to a μ–η^1^:η^2^-CH=C=C(C_5_H_9_) allenyl. The overall structure and bonding values closely resemble those observed in similar diiron and diruthenium allenyl complexes.^57,58,72^ Thus, C(4)–C(5)–C(6) is considerably bent [154.58(18)°], and both C(4)–C(5) [1.365(2) Å] and C(5)–C(6) [1.314(3) Å] contacts indicate a relevant π character. The bridging CO ligand shows an appreciable asymmetry [Ru(1)–C(3) 1.9662(19) Å; Ru(2)–C(3) 2.1974(19 Å] since the bridging allenyl ligand binds Ru(1) in a η^1^ fashion, whereas it is η^3^ coordinated to Ru(2). A lower degree of asymmetry concerns the bridging alkylidene carbon C(4) [Ru(1)–C(4) 2.0407(18), Ru(2)–C(4) 2.1768(17) Å].

**Table 2:** Selected Bond Lengths (Å) and Angles (°) for **3a**

Ru(1)–Ru(2)	2.7768(8)	Ru(2)–C(5)	2.2111(19)
Ru(1)–C(1)	1.885(2)	Ru(2)–C(2)	1.889(2)
Ru(1)–C(3)	1.9662(19)	Ru(2)–C(3)	2.1974(19)
Ru(1)–C(4)	2.0407(18)	Ru(2)–C(4)	2.1768(17)
C(1)–O(1)	1.135(2)	C(2)–O(2)	1.136(2)
C(3)–O(3)	1.156(2)	C(4)–C(5)	1.365(2)
C(5)–C(6)	1.314(3)	C(6)–C(7)	1.513(2)
C(7)–C(8)	1.533(2)	C(8)–C(9)	1.525(3)
C(9)–C(10)	1.534(3)	C(6)–C(10)	1.524(3)
Ru(1)–C(1)–O(1)	178.82(19)	Ru(2)–C(2)–O(2)	177.10(18)
Ru(1)–C(3)–Ru(2)	83.46(7)	Ru(1)–C(4)–Ru(2)	82.29(6)
C(4)–C(5)–C(6)	154.58(18)	C(5)–C(6)–C(7)	127.48(17)
C(5)–C(6)–C(10)	123.22(16)	C(7)–C(6)–C(10)	109.27(15)
C(6)–C(7)–C(8)	104.36(15)	C(7)–C(8)–C(9)	103.10(15)
C(8)–C(9)–C(10)	104.50(15)	C(9)–C(10)–C(6)	103.30(15)

### 2.2 Studies in Aqueous Solutions and Interactions of the Complexes with Reduced Glutathione, l-Cysteine, and Bovine Serum Albumin

In anticipation of biological investigations, the behavior of the synthesized complexes was initially evaluated in aqueous solutions (Table 3). As a starting point, octanol/water partition coefficients (Log *P*_ow_) were determined using a UV–vis method. In general, the cationic allenyl complexes **3a**–**c** display a balanced hydrophilic/hydrophobic character, which is a favorable prerequisite for biological applications, with **3a** being more hydrophilic compared to the estradiol-containing analogue **3b** and the testosterone-derivative **3c**. On the other hand, the neutral compounds **2a**–**c** possess a marked lipophilicity (Log *P*_ow_ > 1.5). Because of their limited solubility in water, the stability of the complexes was gauged in a methanol/water mixture (ca. 1:5 v/v) through UV–vis spectroscopy at various time intervals (Figures S10–S19). In general, cationic complexes **3a**–**c** were substantially more stable than the neutral ones **2a**–**c**. On account of the Log *P*_ow_ and stability data, we decided to focus the biological studies on the allenyl complexes **3a**–**c**, with **2b** selected as a representative species of the series **2a**–**c**. The stability assessment was replicated in the presence of a cell culture medium (DMEM), revealing a gradual degradation of the complexes over an 18 h period, with **3c** emerging as the most robust species (67% residual complex after 18 h). It is presumable that the modification of diruthenium complexes in aqueous solution is facilitated by the displacement of the C=C moiety coordinated to ruthenium by the solvent, in line with previous findings concerning diiron μ-allenyl complexes.^74^ In any case, the released organic species are expected to differ significantly from the alkynol reactants, which reasonably form an irreversible C–C bond in **2a**–**c** and further undergo the loss of the hydroxyl moiety to give **3a**–**c** (Scheme 1).

**Table 3:** Behavior of Diruthenium Complexes in Aqueous Media: Octanol/Water Partition Coefficients (Log *P*_ow_, based on UV–Vis Spectroscopy) at 21 °C and Stability in H_2_O/CH_3_OH and DMEM/CH_3_OH Solutions at Different Times (ca. 5:1 v/v, UV–Vis Analyses)

complex	Log *P*_ow_	residual complex % in H_2_O/CH_3_OH	residual complex % in DMEM/CH_3_OH	time
**2a**	>1.5	97	N.A.	30 min
		91	N.A.	2 h
		23	N.A.	18 h
**2b**	>1.5	69		30 min
			50	2 h
		0	21	18 h
**2c**	>1.5	99	N.A.	30 min
		81	N.A.	2 h
		14	N.A.	18 h
**3a**	–0.54 ± 0.02	96	94	2 h
		78	12	18 h
**3b**	1.24 ± 0.16	99	N.A.	30 min
		92	88	2 h
		43	42	18 h
**3c**	0.89 ± 0.12	94	89	2 h
		73	67	18 h

The ESI-MS spectra of complexes **3a**–**c**, in methanol/water mixture after 24 h, confirmed their substantial stability, each spectrum showing the peak of the cation as the main species (Figures S20–S22). Next, the representative compounds **3a** and **3b** were incubated in methanol/water solutions in the presence of selected biomolecules, i.e., reduced glutathione (GSH) and l-cysteine, respectively. The resulting ESI-MS spectra did not evidence the formation of any product ascribable to the interaction with 10 mM GSH (Figures S23–S24). Note that the selected GSH concentration is expected to mimic the intracellular level in cancer cells.^75,76^ On the other hand, a mixture of nonidentified diruthenium species was produced from the reaction of **3a** with l-cysteine (120 μM^77^), after 24 h incubation (Figure S25). We expanded this study to assess the potential interaction of **3a**–**b** with bovine serum albumin (BSA) as a representative protein model (48 h incubation at 37 °C), using the MALDI-TOF MS technique (Figure S26). Complex **3a** showed a notable affinity toward BSA, and an adduct was detected apparently comprising more than six **3a** cations (resulting in a mass variation of 3434 Da with respect to BSA). On the other hand, the interaction of **3b** with BSA involves the binding of a unit likely originating from the cleavage of the diruthenium structure (leading to a mass variation of 483 Da compared to BSA). Taking into account the uncertainty of the measurement, this unit might correspond to a mononuclear ruthenium complex with the allenylsteroidal moiety coordinated to the {RuCp(CO)_2_} scaffold.

### 2.3 Electrochemistry

The electrochemical behavior of neutral complex **2a** and cationic **3a** was comparatively studied in CH_2_Cl_2_/[N^*n*^Bu_4_]PF_6_ solution, where both complexes are sufficiently soluble. The voltammetric profiles of the compounds (Figure S27) show two irreversible processes, namely, one reduction and one oxidation. According to the nature of the complexes, these processes are shifted toward more cathodic (for the neutral **2a**) or anodic (for the cationic **3a**) potentials (Table 4). While both reduction and oxidation do not seem viable in a biological environment in the case of **3a**, in principle, the oxidation of **2a** could be accessible. In fact, the biologically relevant range of potentials spans from −0.4 to +0.8 V vs SHE.^78,79^ Nevertheless, the low stability of **2a** in aqueous solutions (see above) suggests that its electrochemistry cannot play a significant role.

**Table 4:** Formal Electrode Potentials (*V*, Referenced to Ag/AgCl/KCl and, in Parentheses, to FeCp_2_) for the Redox Transformations Displayed by **2a** and **3a** When Dissolved in CH_2_Cl_2_/[N^*n*^Bu_4_]PF_6_ 0.2 M

complex	reduction	oxidation
**2a**	–1.82^a^ (−2.26)	+0.55^a^ (+0.11)
**3a**	–1.00^a^ (−1.46)	+1.61^a^ (+1.15)

*a* Peak potential value for irreversible processes.

### 2.4 Biological Studies

To the best of our knowledge, biological studies on dimetallacyclopentenone compounds have been limited to iron–platinum complexes, investigated for their interactions with cancer-relevant proteins.^80^ We conducted an in vitro cytotoxicity screening of selected complexes on a panel of nine human cancer cell lines, while the selectivity was evaluated on the normal cell line MRC-5. Cisplatin and RAPTA-C^13^ were employed as standards for comparative purposes. The obtained IC_50_ values are compiled in Table 5, highlighting the promising antiproliferative potential of diruthenium complexes with IC_50_ values generally lower than those measured for cisplatin. Overall, complex **3b** demonstrated the best performance, excelling in terms of both antiproliferative efficacy and selectivity; specifically, the IC_50_ value on the healthy MRC-5 cell line is approximately 2-fold compared to the average IC_50_ values referred to the cancer cell lines. The comparable antiproliferative activity exhibited by **3a**–**c** on the cell lines expressing the estrogen receptors (dominantly A2780, cisplatin-resistant A2780R and MCF-7)^81^ suggests that the presence of the bioactive fragment in **3b** and **3c** does not provide a substantial effect. Nevertheless, it is possible that the estrogen-ligand, which is expected to be gradually released from **3b**–**c** in a physiological solution (see stability studies), may partially contribute to the overall mechanism of antiproliferative effect by exerting its intrinsic biological activity.^82^ Despite the superior lipophilicity, complex **2b** is inactive toward A549, PANC-1, and Caco-2 cells and less cytotoxic than **3a**–**c** against HOS and PC-3 cell lines. The lower performance of **2b** might be associated with its relatively fast degradation (Table 3). The cytotoxicity was then determined on the A2780 cell line at different incubation times (Table 6), pointing out that the activity of diruthenium compounds takes place primarily within the initial 24 h. In fact, the IC_50_ values did not significantly decrease after additional 24 and 48 h. Conversely, a progressive decrease in IC_50_ over 72 h was detected on cisplatin under identical conditions.

**Table 5:** IC_50_ Values (μM) Determined for the Diruthenium Complexes (**2b**, **3a**, **3b** and **3c**) and Reference Compounds (RAPTA-C and Cisplatin) on Human Ovarian Carcinoma (A2780), Cisplatin-Resistant Human Ovarian Carcinoma (A2780cisR), Breast Adenocarcinoma (MCF-7), Human Osteosarcoma (HOS), Human Lung Adenocarcinoma (A549), Human Pancreatic Carcinoma (PANC-1), Human Colorectal Adenocarcinoma (Caco-2), Human Prostate Carcinoma (PC-3), Human Cervical Carcinoma (HeLa), and Normal Human Lung Fibroblast (MRC-5) Cell Lines^a^

	A2780	A2780R	MCF-7	HOS	A549	PANC-1	Caco-2	PC-3	HeLa	MRC-5
**2b**	6.2 ± 1.2	7.3 ± 2.4	19.0 ± 4.5	24.0 ± 3.8	>50	>50	>50	36.0 ± 4.1	5.5 ± 0.9	>50
**3a**	4.2 ± 0.9	6.4 ± 1.9	16.2 ± 1.7	14.6 ± 0.5	25.3 ± 1.9	28.4 ± 3.9	>50	22.2 ± 2.4	17.5 ± 2.9	38.3 ± 3.9
**3b**	3.4 ± 0.6	4.6 ± 1.3	11.6 ± 1.5	12.6 ± 0.5	16.1 ± 1.3	19.8 ± 2.3	36.0 ± 2.7	13.4 ± 1.8	12.5 ± 1.1	25.9 ± 2.9
**3c**	6.3 ± 1.3	11.7 ± 2.4	22.0 ± 4.0	17.7 ± 2.8	20.7 ± 1.4	30.0 ± 0.6	42.8 ± 0.8	19.6 ± 3.7	16.3 ± 1.3	21.5 ± 4.0
RAPTA-C	>50	>50	>50	>50	>50	>50	>50	>50	>50	>50
cisplatin	15.2 ± 1.1	40.0 ± 3.9	28.4 ± 2.7	26.3 ± 3.3	39.2 ± 3.1	>50	>50	>50	30.7 ± 0.6	>50

a Incubation time = 24 h. The values are presented as the mean ± the standard deviation (SD).

**Table 6:** IC_50_ Values (μM) Determined for Diruthenium Complexes and Reference Compounds on the A2780 Human Ovarian Carcinoma Cell Line (A2780) Following Varying Incubation Times^a^

	**incubation time (A2780 cell line)**		
	24 h	48 h	72 h
**2b**	5.8 ± 0.4	4.7 ± 0.8	4.2 ± 0.6
**3a**	3.8 ± 0.7	3.2 ± 0.2	3.1 ± 0.2
**3b**	4.0 ± 0.7	3.0 ± 0.3	2.8 ± 0.2
**3c**	4.7 ± 1.0	5.1 ± 0.7	6.8 ± 1.3
RAPTA-C	>50	>50	>50
cisplatin	18.0 ± 3.7	12.4 ± 1.8	5.8 ± 1.8

a The values are presented as the mean ± standard deviation (SD).

#### 2.4.1 Cellular Uptake

**Figure 4:**
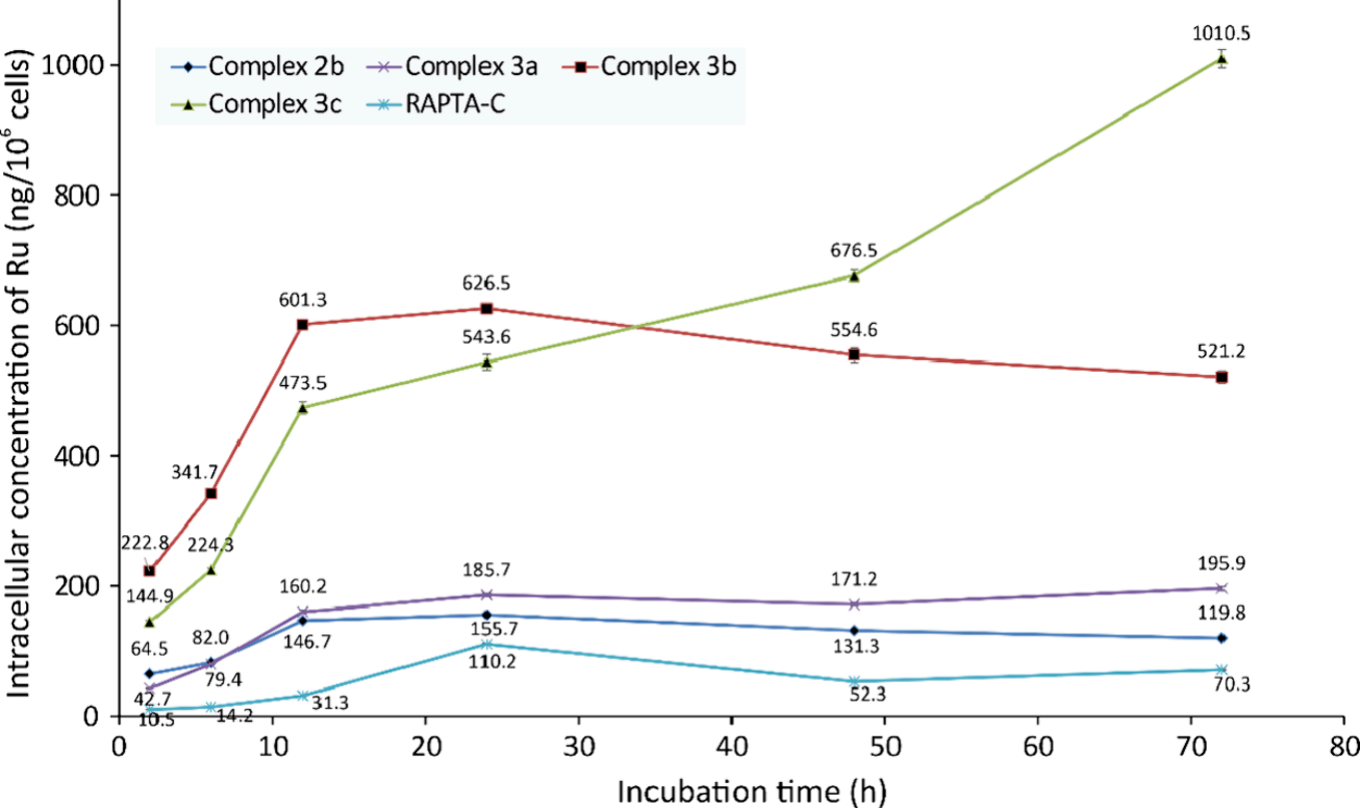
Ruthenium cellular uptake in A2780 cells after different times of incubation at different IC_50_ concentrations.

The uptake in A2780 cells was determined for complexes **2b**, **3a**, **3b,** and **3c** at the respective IC_50_ concentrations at different times by ICP-MS measurement of the intracellular ruthenium level (Figure 4). RAPTA-C, for which a negligible in vitro internalization in cancer cells is documented,^11,83^ was employed as a reference.

In general, the internalization of ruthenium species in the cancer cells increases during the initial 12 h and then remains approximately unchanged, presumably due to the occurrence of disassembly of the organometallic scaffold as suggested by the stability studies. The further increase of cellular uptake observed for **3c** after 12 h may be ascribable to the formation of ruthenium-containing fragmentation products with a specific ability to cross the cell membrane. The degree of internalization in the first 12 h follows the order: RAPTA-C < **2b** < **3a** < **3c** < **3b**. Concerning allenyl complexes **3a**–**c**, this order is in alignment with the Log *P*_ow_ values and the cytotoxicity data. Conversely, the reduced intracellular accumulation of *lipophilic* compound **2b** reflects its minor antiproliferative efficacy.

#### 2.4.2 Cell Cycle Analysis

**Figure 5:**
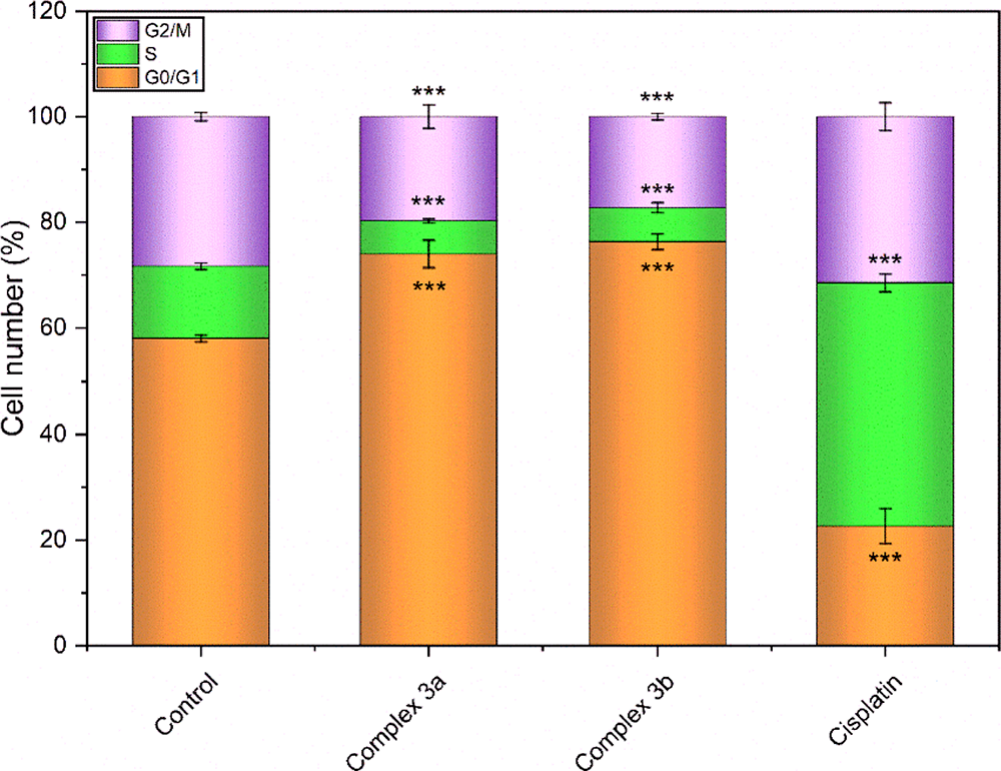
Effect of cationic diruthenium complexes **3a** and **3b** and cisplatin on the cell cycle in A2780 cells. The results labeled with *** denote a statistically significant distinction between the designated cell population and the control group at the significance level of *p* < 0.001.

The performant cationic complexes **3a** and **3b** underwent a targeted study to assess their influence on the cell cycle of A2780 cells. Both compounds showed a profound effect (Figure 5), characterized by strong depression of the cell number in the synthetic (S) phase and second gap and mitotic phases (G2/M) of the cycle, thereby increasing the population of nondividing cells (in the G0/G1 phase). Cisplatin was analyzed as a reference drug, known for its primary mechanism of action involving the covalent alteration of DNA,^84^ thereby halting cell progression in the synthetic phase of the cell cycle.^85^ On the other hand, the studied diruthenium complexes probably act at different sites and through different modes of action, possibly associated with the lowering of the metabolic activity and consequently the dividing capacity of the target cells. Numerous previous studies (for more details, see e.g., Zaki et al.^86^) pointed out the capability of organo-ruthenium complexes to affect the metabolic activity of cancer cells through the interference with several metabolic pathways, such as iron and ATP metabolism,^87^ calcium metabolism in mitochondria^88,89^ and various enzymatic routes.

#### 2.4.3 Induction of Apoptosis and Autophagy

**Figure 6:**
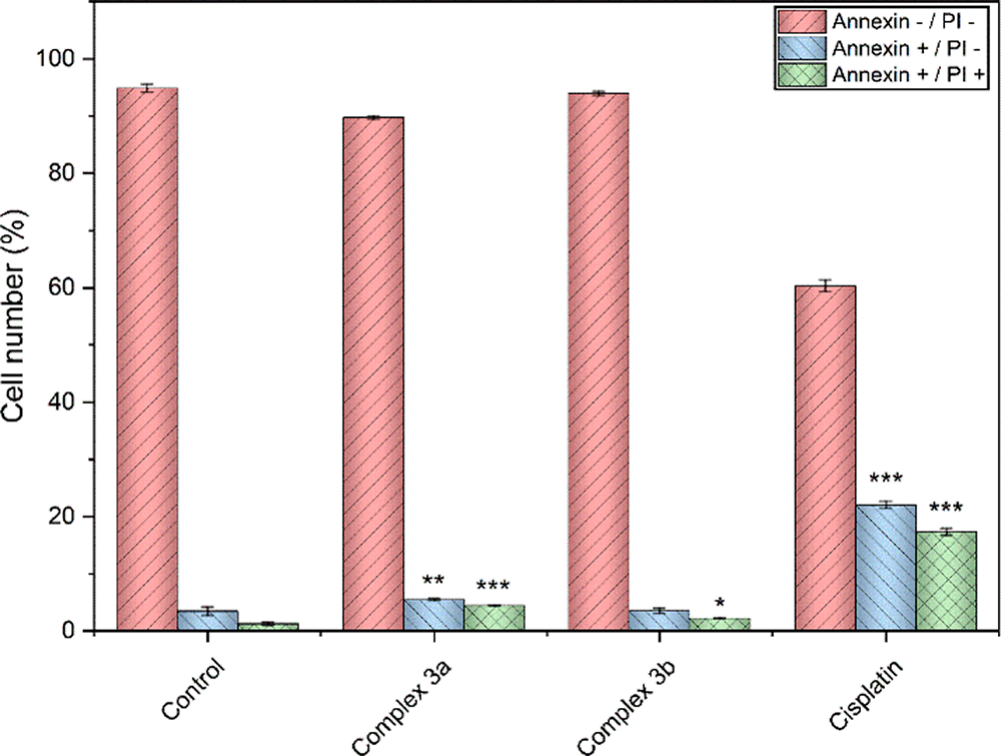
Effect of diruthenium complexes **3a** and **3b** and cisplatin on apoptosis in A2780 cells. The results marked with an asterisk represent a statistically significant difference between the specific population of cells and the control group at *p < 0.05, **p < 0.01, and ***p < 0.001 levels.

The proapoptotic effects of **3a**–**b** and cisplatin were assessed on A2780 cells through flow cytometry using Annexin V/propidium iodide (PI) staining (Figure 6). The findings indicate a relatively low effect on the raising of the cell count during the initial phases of apoptosis (Annexin positive/PI negative) and an even lower effect in the advanced stages of apoptosis (Annexin positive/PI positive). Although the effect provided by **3a** is quantitatively stronger than that of **3b**, overall the proapoptotic activities of **3a** and **3b** are much less effective than that of cisplatin, which elicited apoptotic changes in ca. 40% of the cells.

**Figure 7:**
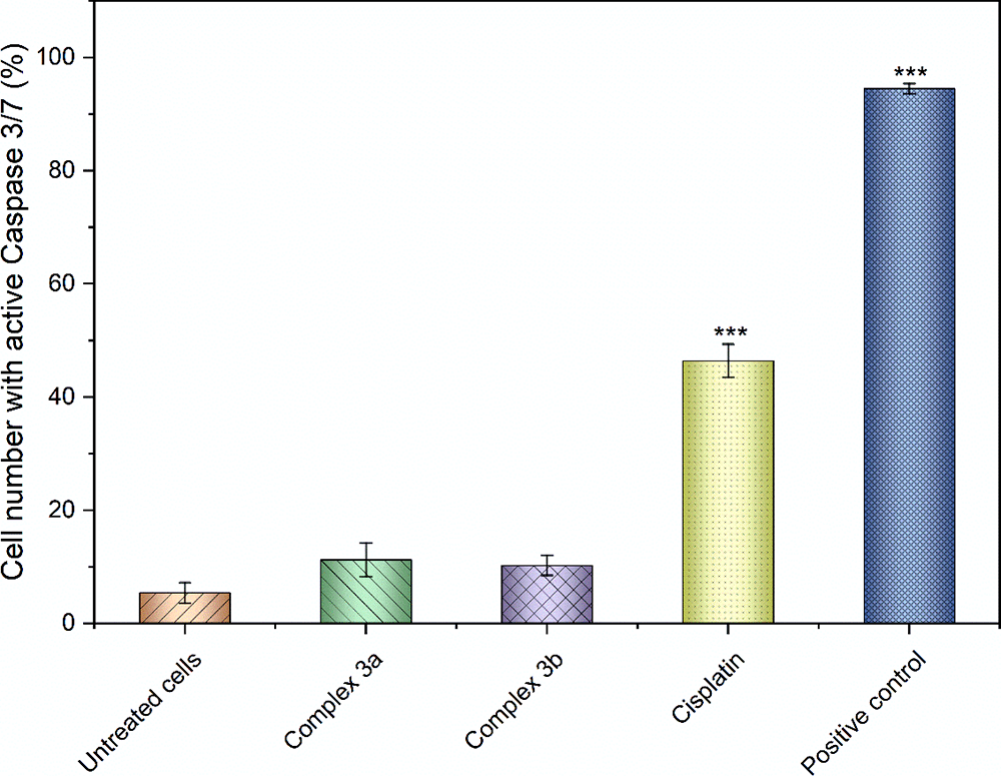
Stimulation of caspases 3/7 in A2780 cells induced by **3a** and **3b** and cisplatin. The results marked with an asterisk represent a statistically significant difference between the specific population of cells and the control group at ***p < 0.001 level.

The results of Annexin V/PI staining were confirmed by the selective flow cytometric identification of the cells with active executioner caspase 3/7, which are activated at the late stages of apoptosis and induce the degradation of intracellular matrix and cytoskeleton and DNA fragmentation (Figure 7). As a matter of fact, **3a**–**b** negligibly increased the cell population with the activated caspase 3/7, unlike cisplatin for which apoptosis induction represents a major mode of action.^84^

**Figure 8:**
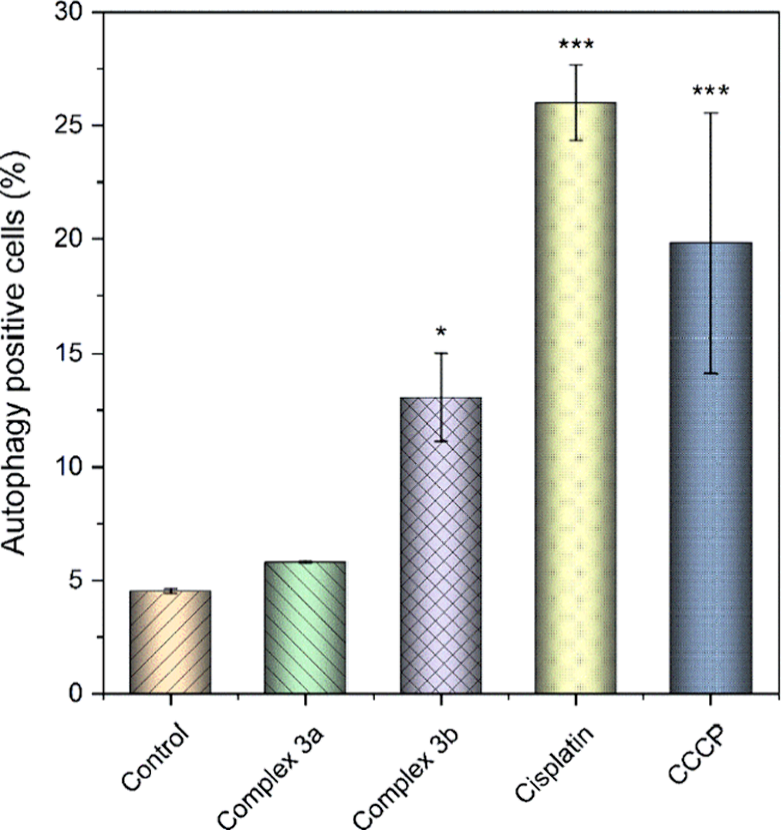
Activation of Autophagy in A2780 cells induced by **3a** and **3b** and cisplatin. The results marked with an asterisk represent a statistically significant difference between the specific population of cells and the control group at *p < 0.05 and ***p < 0.001 levels.

Autophagy represents another type of cell death, usually associated with the physiological response of the cells to uncontrollable cellular stress and, often, with cancer tissue survival. The autophagy level (Figure 8) was significantly enhanced in cisplatin-treated cells, but only moderately in the cells treated with **3b**, whereas the effect of **3a** was not appreciable. Nevertheless, the induction of autophagy by **3b** may be triggered by the release of the bioactive loading and could considerably contribute to the antiproliferative effect of this complex. A similar behavior was previously reported for an estradiol-platinum(II) complex.^90^

#### 2.4.4 Intracellular Oxidative Stress and Mitochondrial Membrane Damage

**Figure 9:**
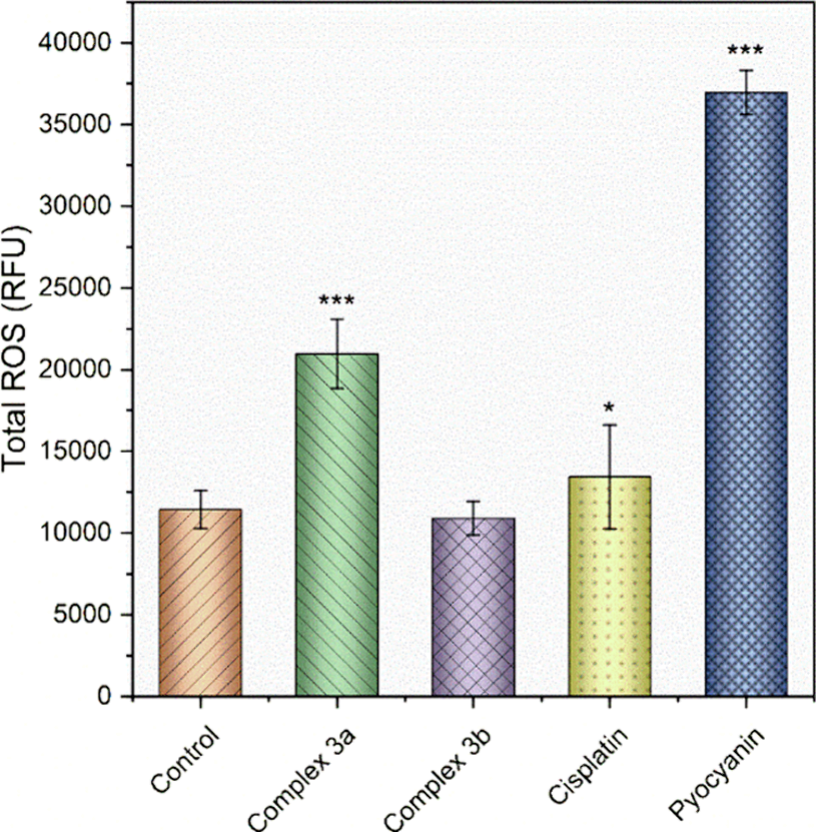
Induction of oxidative stress in A2780 cells by treatment with **3a** and **3b** and the drug cisplatin. As a positive control, the well-known ROS inducer pyocyanin was used in 100 μM concentration. The results marked with an asterisk represent a statistically significant difference between the specific population of cells and the control group at *p < 0.05 and ***p < 0.001 levels.

Due to the availability of adjacent, relatively stable oxidation states, associated with a versatile redox chemistry, transition metal complexes may be prone to induce intracellular oxidative stress.^91^ This may occur via direct production of an excess of *reactive oxygen species* (ROS) or by depleting the capacity of natural intracellular defenses against oxidative stress (e.g., by direct inhibition of antioxidant enzymes or inhibition of small antioxidant molecules such as GSH). Thus, incubation of A2780 cells with representative complex **3a** resulted in a noteworthy elevation of intracellular ROS levels (Figure 9); interestingly, no appreciable effect was detected for **3b** under the same experimental conditions.

**Figure 10:**
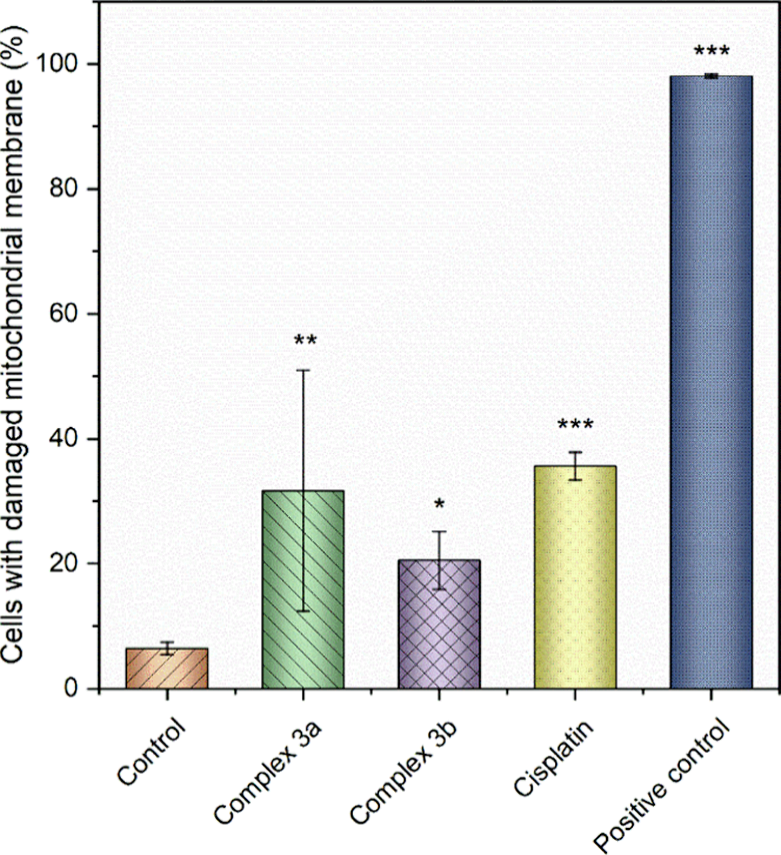
Effect of **3a** and **3b** and cisplatin on the mitochondrial membrane integrity in A2780 cells. The results marked with an asterisk represent a statistically significant difference between the specific population of cells and the control group at: *p < 0.05, **p < 0.01, and ***p < 0.001 levels.

One of the consequences of localized intracellular overproduction of ROS is the damaging of cell membrane structures.^92^ Concerning mitochondrial membranes, this phenomenon depletes oxidative metabolism and cellular respiration. An additional plausible consequence of mitochondrial membrane damage is the liberation of cytochrome c from the inner mitochondrial membrane, leading to an intrinsic pathway of apoptosis by apoptosome sequestration and subsequent activation of executioner caspases. Both cisplatin and the diruthenium complexes **3a** and **3b** led to a notable augmentation in the cell count with damaged mitochondrial membranes (Figure 10). Consequently, this biological process could potentially contribute to the mechanism of action of diruthenium complexes. According to literature findings,^93^ also the elimination of carbon monoxide from the complexes, as indirectly outlined by the stability studies (see above), may contribute to intracellular metabolic stress, although additional targeted investigations are needed to confirm this hypothesis.

#### 2.4.5 Concluding Remarks

Ruthenium complexes are pivotal in the quest for novel and efficacious anticancer metallodrugs, with the aim of surpassing the constraints of currently used platinum drugs in chemotherapy. So far, research efforts have been primarily focused on mononuclear complexes based on the ruthenium-arene framework. Meanwhile, diruthenium complexes, despite their potential benefits stemming from metal–metal cooperative phenomena, have received less attention. In this work, we present the synthesis and evaluation of the anticancer capabilities of novel diruthenium complexes featuring diverse bridging hydrocarbyl ligands. These complexes represent rare derivatives of the commercial [Ru_2_Cp_2_(CO)_4_] evaluated in the biological field. We provide evidence that a simple synthetic protocol enables the incorporation of various substituents on the bridging ligand, including steroidal fragments, thus modulating the physicochemical characteristics and cytotoxicity of the complexes. Cationic diruthenium μ-allenyl complexes were revealed to be promising, in that they display a strong antiproliferative potency against a spectrum of cancer cell lines (typically exhibiting IC_50_ values lower than those associated with cisplatin). This potency is accompanied by a propensity for selectivity. Targeted experiments suggest a multimodal mechanism of action, which seems sensitive to local structural variations. We recognized, for the most active compounds, a significant influence on the cell cycle and the interference with the cellular metabolism at different levels, resulting in the enhancement of ROS production, the depletion of mitochondrial membrane potential, and the induction of autophagy. It is likely that the activity is ascribable, at least in part, to the modification of the complexes in the biological environment, including the gradual release of ligands (estradiol-molecules and CO). It is also conceivable that protein binding plays some role, as suggested by ESI-MS interaction studies with a model protein. Nevertheless, an accelerated decomposition, as evidenced for neutral diruthenacyclopentenone complexes, is detrimental to the activity. Since the here proposed synthetic strategy possesses a general character, a broad scope is offered for the future development of diruthenium allenyl complexes with optimized properties and activities.

## 3 Experimental Section

### 3.1 Synthesis and Structural Characterization of Diruthenium Complexes

#### 3.1.1 General Details

Reagents and solvents were obtained from different sources (Merck, Alfa Aesar, TCI Chemicals or Strem) and were of the utmost purity commercially attainable. Complex **1** was synthesized using the literature procedure.^55^ The reactions were performed within a N_2_ atmosphere by employing conventional Schlenk techniques. After isolation, the products were stored in ambient air conditions. Dichloromethane and tetrahydrofuran were dried using the solvent purification system mBraun MB SPS5, whereas acetonitrile was distilled over calcium hydride. Infrared spectra of solutions were acquired by using a CaF_2_ liquid transmission cell (2300–1500 cm^–1^) through a PerkinElmer Spectrum 100 FT-IR spectrometer. Spectragryph software was used for processing the infrared spectra.^94^ NMR spectra were recorded on a Jeol JNM-ECZ500R instrument equipped with a Royal HFX Broadband probe (at a temperature of 298 K, unless otherwise specified). Chemical shifts are expressed in parts per million and referenced to the residual solvent peaks.^95^ NMR spectra were assigned with the assistance of ^1^H–^13^C (*gs*-HSQC and *gs*-HMBC) correlation experiments.^96^ NMR signals related to secondary isomeric forms (where detectable) are denoted in italics. Elemental analyses were conducted by using a Vario MICRO cube instrument (Elementar). ESI-MS analyses were performed with the Bruker amaZon SL ion-trap mass spectrometer using the ESI ion source in positive ionization mode, except for experiments with GSH which were performed on a Shimadzu Liquid Chromatograph Mass Spectrometer LCMS-8050 using the ESI-8050 ionization unit in positive ionization mode. MALDI-TOF MS analyses were performed using a Microflex LRF20 (Bruker Daltonik, Germany) mass spectrometer. ICP-MS analyses were performed on an Agilent 7700× mass spectrometer with the external calibration, using the Merck Transition metal mix 3 for ICP standard solution.

#### 3.1.2 Synthesis and Characterization of Diruthenacyclopentenone Complexes (**2a**–**c**)

#### 3.1.3 General Procedure

A solution of (ca. 0.15 mmol) in toluene (30 mL) was treated with an excess (ca. 5 equiv) of the selected propargyl alcohol. The resultant mixture was stirred at reflux temperature for a duration of 1 h. The resulting solution was then permitted to cool to room temperature and the solvent was subsequently evaporated under reduced pressure at 50 °C. The residue was subjected to washing with pentane (3 × 20 mL) and then dried under vacuum.

##### 3.1.3.1 [Ru_2_Cp_2_(CO)_2_{μ–η^1^:η^3^-CH=C(1-cyclopentanol)C(=O)}], **2a**

**Chart 1:**
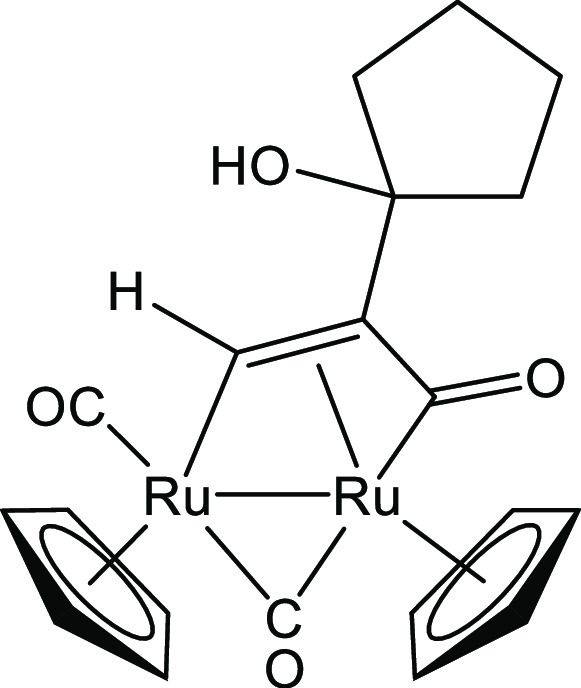
Structure of **2a**

From **1** (78 mg, 0.13 mmol) and 1-ethynylcyclopentanol (0.11 mL, 0.96 mmol) (Chart 1). Red solid, yield 66 mg (96%). Anal. calcd for C_20_H_20_O_4_Ru_2_: C, 45.62; H, 3.83; found: C, 45.59; H, 3.87. IR (CH_2_Cl_2_): υ̌/cm^–1^ = 1977s (CO); 1801m (μ-CO); 1739w (C=O). ^1^H NMR (acetone-d_6_): δ 10.87 (s, 1 H, μ-CH=); 5.55, 5.25 (s, 10 H, Cp); 3.58 (s, br, OH); 1.98–1.94, 1.82–1.62, 1.45–1.41 (m, 8 H, CH_2_). ^13^C{^1^H} NMR (acetone-d_6_): δ 237.1 (μ-CO); 220.6 (C=O); 201.2 (CO); 151.9 (μ-CH=); 90.4, 88.1 (Cp); 81.7 (COH); 53.1 (CH=C); 41.8, 40.9 (CHCH_2_); 25.4, 24.6 (CH_2_). ESI-MS: *m*/*z* = 528.99 (theoretical for [M + H]^+^: *m*/*z* = 528.95).

##### 3.1.3.2 [Ru_2_Cp_2_(CO)_2_{μ–η^1^:η^3^-CH=C(17α-estradiol)C(=O)}], **2b**

**Chart 2:**
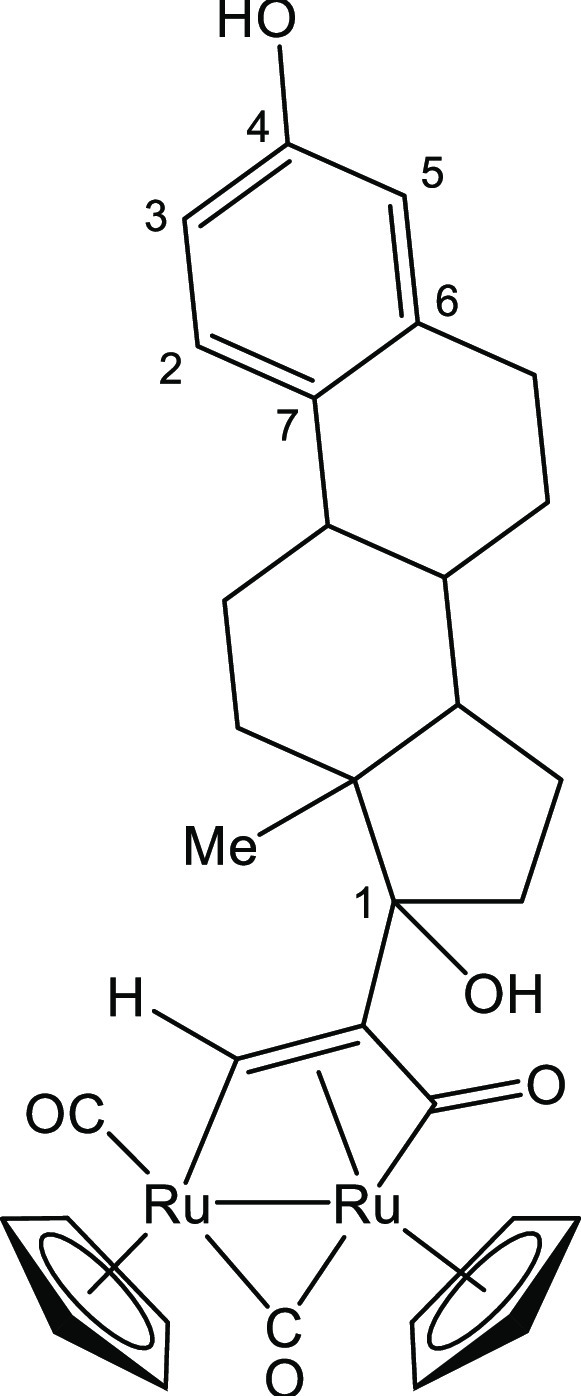
Structure of **2b**

From **1** (83 mg, 0.14 mmol) was 17α-ethynylestradiol (296 mg, 1.00 mmol) (Chart 2). Orange solid, yield 94 mg (94%). Anal. calcd for C_33_H_34_O_5_Ru_2_: C, 55.61; H, 4.81; found: C, 55.69; H, 4.87. IR (CH_2_Cl_2_): υ̌/cm^–1^ = 1976vs (CO), 1803s (μ-CO), 1751w (C=O). ^1^H NMR (acetone-d_6_): δ 10.70 (s, 1 H, μ-CH=); 7.93 (br, 1H, C^4^–OH); 7.11 (d, 1 H, ^3^*J*_HH_ = 8.7 Hz, C^2^H); 6.60 (dd, 1 H, ^3^*J*_HH_ = 8.7 Hz, ^4^*J*_HH_ = 2.8 Hz, C^3^H); 6.53 (d, 1 H, ^4^*J*_HH_ = 2.8 Hz, C^5^H); 5.55, 5.27 (s, 10 H, Cp); 3.46 (s, br, C^1^–OH); 2.37–2.28, 2.25–2.14, 2.00–1.86, 1.69–1.60 (m, 15 H, CH + CH_2_); 1.02 (s, 3 H, Me). ^13^C{^1^H} NMR (acetone-d_6_): δ/ppm = 237.1 (μ-CO); 219.6 (C=O); 201.5 (CO); 156.0 (C^4^); 154.1 (μ-CH=); 138.5 (C^6^); 132.0 (C^7^); 127.2 (C^2^); 116.0 (C^5^); 113.7 (C^3^); 90.3, 88.2 (Cp); 84.8 (C^1^); 49.8 (CH=C); 79.8, 74.5, 50.4, 48.7, 44.7, 40.9, 40.7, 40.0, 36.7, 34.8, 33.8, 30.4, 28.3, 27.6, 27.4, 24.0, 23.5, 15.8 (C + CH + CH_2_); 13.3 (Me). ESI-MS: *m*/*z* = 736.18 (theoretical for [M + Na]^+^: *m*/*z* = 737.04). Crystals of **2b** appropriate for X-ray analysis were obtained by permitting slow diffusion of hexane into a dichloromethane solution at room temperature.

##### 3.1.3.3 [Ru_2_Cp_2_(CO)_2_{μ–η^1^:η^3^-CHC(17-testosterone)C(O)}], **2c**

**Chart 3:**
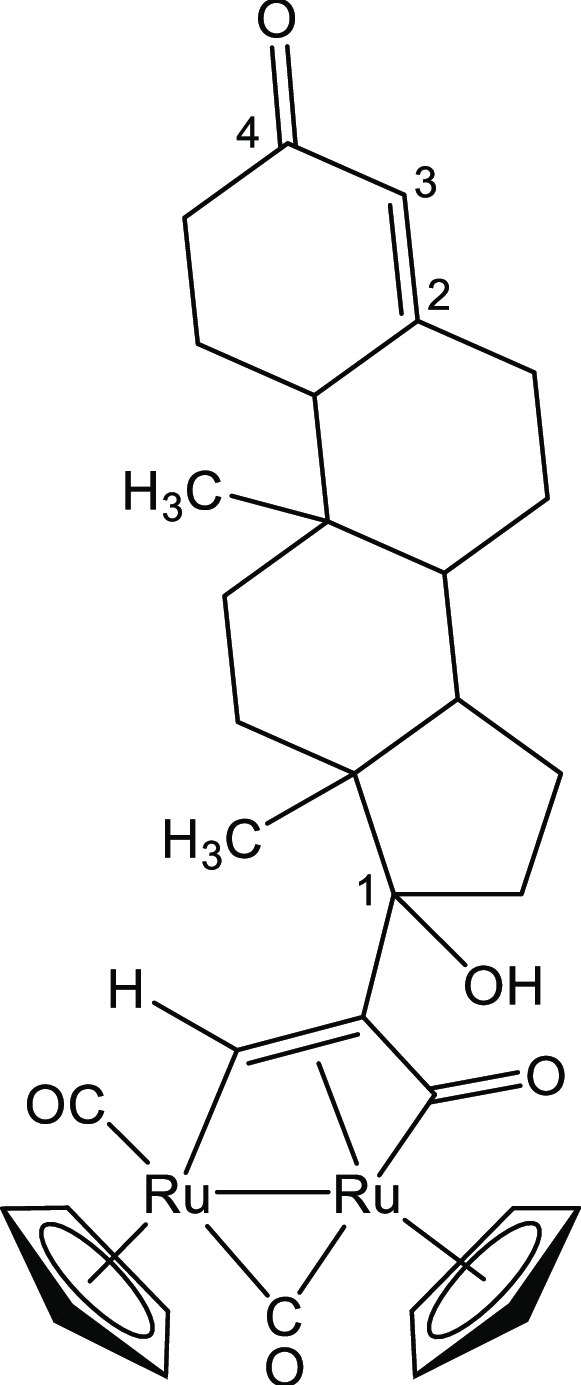
Structure of **2c**

From **1** (71 mg, 0.12 mmol) and 17-ethynyltestosterone (244 mg, 0.78 mmol) (Chart 3). Orange solid, 84 mg (96%). Anal. calcd for C_34_H_38_O_5_Ru_2_: C, 56.03; H, 5.26; found: C, 56.12; H, 5.14. IR (CH_2_Cl_2_): υ̌/cm^–1^ = 1976vs (CO), 1803s (μ-CO), 1751w (C=O). ^1^H NMR (acetone-d_6_): δ 10.72 (s, 1 H, μ-CH=); 5.53 (s, 1 H, C^3^H); 5.51, 5.22 (s, 10 H, Cp); 3.41 (s, br, C^1^–OH); 2.38–2.28, 2.21–2.12, 1.66–1.57, 1.51–1.41 (m, 19 H, CH + CH_2_); 1.19, 1.00 (s, 6 H, Me). ^13^C{^1^H} NMR (acetone-d_6_): δ 237.1 (μ-CO); 219.6 (RuC=O); 201.5 (CO); 198.2 (C^4^=O); 171.2 (C^2^), 153.9 (μ-CH=); 124.3 (C^3^); 90.3, 88.2 (Cp); 84.7 (C^1^); 49.4 (CH=C); 55.9, 55.1, 50.9, 49.3, 47.5, 39.9, 39.3, 37.2, 37.1, 36.8, 36.7, 34.6, 34.5, 33.2, 32.8, 32.6, 24.8, 24.3, 23.8, 21.9, 21.5 (C + CH + CH_2_); 17.8, 15.7 (Me). ESI-MS: *m*/*z* = 731.50 (theoretical for [M + H]^+^: *m*/*z* = 731.09).

#### 3.1.4 Synthesis of Diruthenium Allenyl Complexes (**3a**–**c**)

##### 3.1.4.1 General Procedure

A solution of the selected complex (ca. 0.10 mmol) in CH_2_Cl_2_ (15 mL) was treated with HBF_4_·Et_2_O (1 equiv) which was added dropwise at room temperature; stirring was maintained for 15 min. Subsequently, H_2_O (ca. 5 mL) was added, and the resultant mixture was vigorously agitated. The organic phase was isolated and desiccated under a reduced pressure. The resulting solid residue was dissolved in CH_2_Cl_2_ (4 mL), and precipitation occurred upon the addition of diethyl ether (60 mL). The resultant solid was subjected to washing with diethyl ether (3 × 15 mL) and then dried under vacuum.

##### 3.1.4.2 [Ru_2_Cp_2_(CO)_3_{μ–η^1^:η^2^-CH=C=(cyclopentylidene)}]BF_4_, **3a**

**Chart 4:**
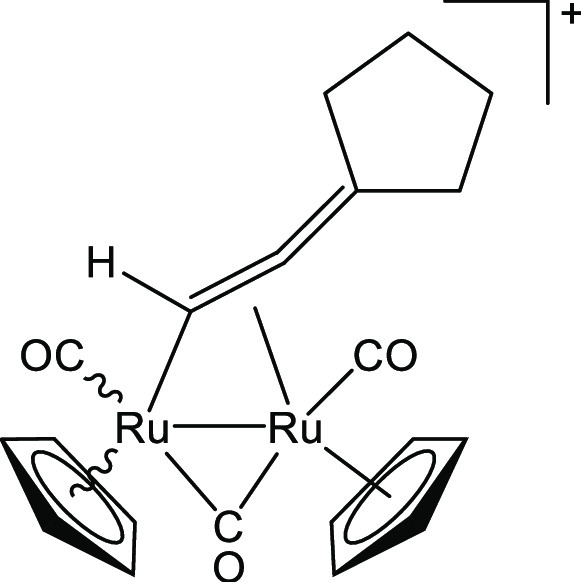
Structure of the Cation of **3a**

From **2a** (58 mg, 0.110 mmol) and HBF_4_·Et_2_O (16 μL, 0.12 mmol) (Chart 4). Dark-yellow solid, yield 56 mg (85%). Anal. calcd for C_20_H_19_BF_4_O_3_Ru_2_: C, 40.28 H, 3.21; found: C, 40.20; H, 3.18. IR (CH_2_Cl_2_): υ̌/cm^–1^ = 2039vs (CO), 2018m–s (CO), 1871m (μ-CO). ^1^H NMR (acetone-d_6_, 253 K): δ 10.80, 9.84 (q, ^5^*J*_HH_ = 2.8 Hz, 1 H, μ-CH=); 6.12, 5.98, *5.95, 5.80* (s, 10 H, Cp); *2.88–2.77*, 2.66–2.58, 2.33–2.27, 1.86–1.68 (m, 8 H, CH_2_). Isomer ratio (cis/trans) ≈ 4. ESI-MS: *m*/*z* = 510.93 (theoretical for [M]^+^: *m*/*z* = 510.94). Crystals of **3a** appropriate for X-ray analysis were obtained by permitting slow diffusion of diethyl ether into a dichloromethane solution at room temperature.

##### 3.1.4.3 [Ru_2_Cp_2_(CO)_3_{μ–η^1^:η^2^-CH=C=(estradiolylidene)}]BF_4_, **3b**

**Chart 5:**
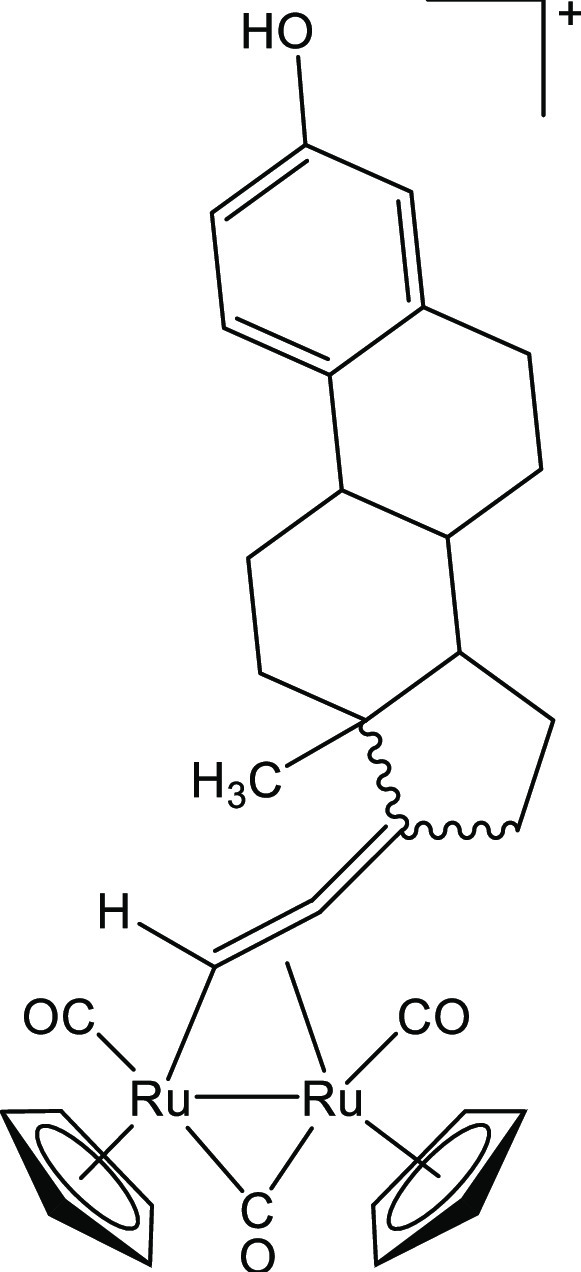
Structure of the Cation of **3b**

From **2b** (59 mg, 0.10 mmol) and HBF_4_·Et_2_O (14 μL, 0.10 mmol) (Chart 5). Orange solid, yield 68 mg (87%). Anal. calcd for C_33_H_33_BF_4_O_4_Ru_2_: C, 50.65; H, 4.25; found: C, 50.69; H, 4.30. IR (CH_2_Cl_2_): υ̌/cm^–1^ = 2037vs (CO), 2011m–sh (CO), 1868m (μ-CO). ^1^H NMR (acetone-d_6_, 253 K): δ ppm = 10.98, 10.90 (t, 1 H, ^5^*J*_HH_ = 3.0 Hz, μ-CH=); 8.29 (s, 1 H, OH); 7.12, 7.09 (d, 1 H, ^3^*J*_HH_ = 8.3 Hz, arom CH); 6.62–6.58, *6.54–6.51* (m, 2 H, arom CH); 6.19, *6.18, 6.01*, 5.99 (s, 10 H, Cp); 2.90–2.55, 1.60–1.44 (m, 15 H, CH + CH_2_); 1.05, 0.88 (s, 3 H, Me). Isomer ratio (*E*/*Z*) ≈ 2.6. ESI-MS: *m*/*z* = 696.12 (theoretical for [M]^+^: *m*/*z* = 696.03).

##### 3.1.4.4 [Ru_2_Cp_2_(CO)_3_{μ–η^1^:η^2^-CH=C=(testosteronylidene)}]BF_4_, **3c**

**Chart 6:**
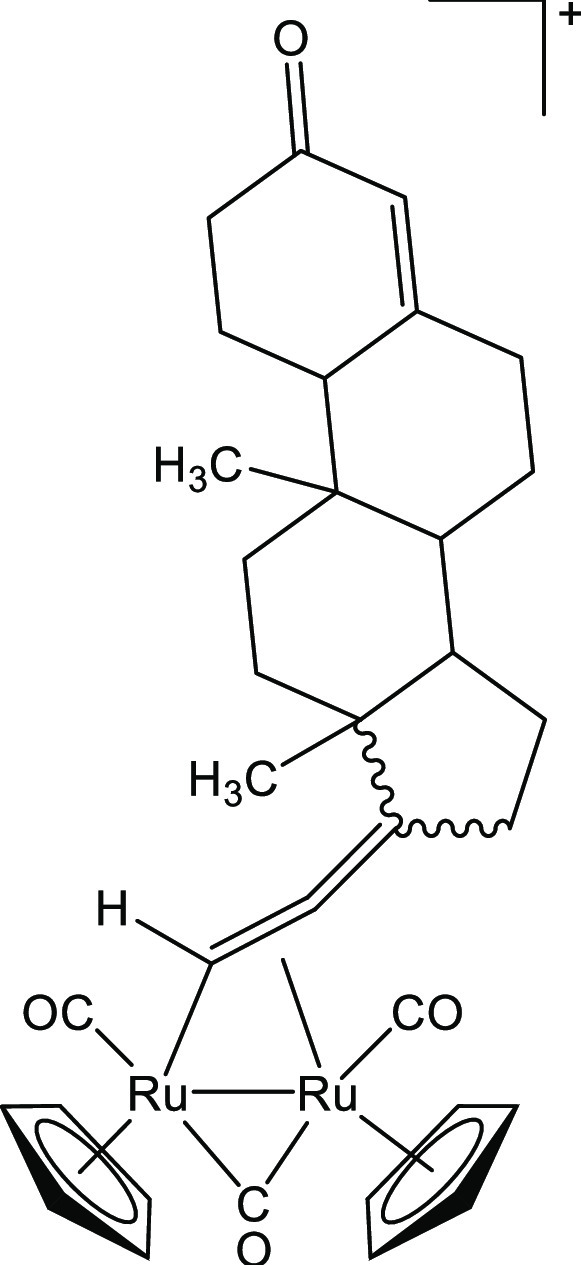
Structure of the cation of **3c**

From **2c** (80 mg, 0.11 mmol) and HBF_4_·Et_2_O (15 μL, 0.11 mmol) (Chart 6). Yellow solid, yield 82 mg (93%). Anal. calcd for C_34_H_37_BF_4_O_4_Ru_2_: C, 51.14; H, 4.67; found: C, 51.10; H, 4.63. IR (CH_2_Cl_2_): υ̌/cm^–1^ = 2037vs (CO), 2011m–sh (CO), 1869m (μ-CO), 1666m (C=O). ^1^H NMR (acetone-d_6_, 253 K): δ 10.93, 10.88 (t, 1 H, ^5^*J*_HH_ = 3.1 Hz, μ-CH=); 6.16, 6.15, 5.99, 5.97 (s, 10 H, Cp); 5.62, 5.61 (s, 1 H, O=CCH=); 2.66–2.37, 2.31–2.14, 1.93–1.86, 1.70–1.59 (m, 19 H, CH + CH_2_); 1.25, 1.23, 1.07, 0.89 (s, 6 H, Me). Isomer ratio (*E*/*Z*) = 4. ESI-MS: *m*/*z* = 713.25 (theoretical for [M]^+^: *m*/*z* = 713.08).

#### 3.1.5 X-ray Crystallography

Crystallographic data for **2b·H**_**2**_**O** and **3b** are listed in Table 7. Experimental data were collected on a Bruker APEX II diffractometer equipped with a PHOTON2 detector, utilizing Mo–Kα radiation. The structures were initially resolved through direct methods and subsequently refined by full-matrix least-squares involving all the collected data using *F*^2^.^97^ Hydrogen atoms were positioned at computed coordinates and subjected to refinement using a riding model except for those connected to the H_2_O molecule of **2b·H**_**2**_**O**. The latter was identified in the Fourier Difference Map and then refined isotropically using the 1.5-fold *U*_iso_ parameter of the parent O atom.

**Table 7:** Crystal Data and Measurement Details for **2b·H**_**2**_**O** and **3a**

	2b·H_2_O	3a
formula	C_33_H_36_O_6_Ru_2_	C_20_H_19_BF_4_O_3_Ru_2_
FW	730.76	596.30
*T*, K	100(2)	100(2)
λ, Å	0.71073	0.71073
crystal system	orthorhombic	monoclinic
space group	P*2*_*1*_*2*_*1*_*2*_*1*_	*P2*_*1*_*/n*
*a*, Å	7.6967(8)	12.341(4)
*b*, Å	13.5569(14)	10.298(4)
*c*, Å	27.138(3)	15.762(5)
β, °	90	92.172(15)
cell volume, Å^3^	2831.7(5)	2001.7(12)
*Z*	4	4
*D*_c_, g·cm^–3^	1.714	1.979
μ, mm^–1^	1.113	1.565
*F*(000)	1480	1168
crystal size, mm	0.15 × 0.12 × 0.07	0.24 × 0.22 × 0.19
θ limits, °	1.679–25.047	2.058–26.990
reflections collected	18923	30377
independent reflections	5006 [*R*_*int*_ = 0.1347]	4357 [*R*_*int*_ = 0.0278]
data/restraints/parameters	5006/383/379	4357/0/271
goodness on fit on *F*^2^^a^	1.184	1.083
*R*_1_ (*I* > 2σ(*I*))^b^	0.0838	0.0174
*wR*_2_ (all data)^c^	0.1694	0.0417
largest diff. peak and hole, e Å^–3^	1.560/–1.956	0.757/–0.491

*a* Goodness on fit on *F*^2^ = [Σ*w*(*F*_O_^2^ – *F*_C_^2^)^2^/(*N*_ref_*–* N_param_)]^1/2^, where *w* = 1/[σ^2^(*F*_O_^2^) + (*aP*)^2^ + *bP*], where *P* = (*F*_O_^2^ + 2*F*_C_^2^)/3; *N*_ref_ = number of reflections employed in the refinement; *N*_param_ = number of parameters refined.

*b* *R*_1_ = Σ||*F*_O_| – |*F*_C_||/Σ|*F*_O_|.

*c* *wR*_2_ = [Σ*w*(*F*_O_^2^ – *F*_C_^2^)^2^/Σ*w*(*F*_O_^2^)^2^]^1/2^, where *w* = 1/[σ^2^(*F*_O_^2^) + (*aP*)^2^ + *bP*], where *P* = (*F*_O_^2^ + 2*F*_C_^2^)/3.

### 3.2 Behavior in Aqueous Media

#### 3.2.1 Determination of Partition Coefficients (Log *P*_ow_)

Partition coefficients (*P*_ow_), defined by the formula *P*_ow_ = *c*_org_/*c*_aq_, wherein *c*_org_ and *c*_aq_ denote the molar concentrations of the specific compound in octanol and aqueous phases, respectively, were obtained using the shake-flask method with UV–vis measurements, following a previously described procedure (Table 3).^98^ All the operations were carried out at 21 ± 1 °C. The wavelength corresponding to the maximum absorption of each compound within the range 350–430 nm was employed for quantifying using UV–vis techniques.

#### 3.2.2 Stability in Aqueous Solutions (UV–Vis)

Solutions with an approximate concentration of 10^–5^ M diruthenium complexes, formulated in a mixture of MeOH and H_2_O (about 1:5 v/v), underwent UV–vis spectroscopic analysis immediately after sample preparation (*t*_0_) and after storage for 4 h at room temperature. The percentage of remaining complex in the solution was calculated by considering the change in absorbance at the characteristic wavelength. The identical procedure was employed to evaluate stability in MeOH and DMEM (about 1:5 v/v) solutions. DMEM stands for the cell culture medium (containing 1000 mg/L glucose and l-glutamine, excluding sodium bicarbonate and phenol red; reference D2902-Merck).

#### 3.2.3 Stability in Aqueous Solutions and Interactions with GSH and l-Cysteine (ESI-MS)

Methanol solutions of diruthenium complexes (**3a** or **3b**, 10 μM) were mixed with an equal volume of an aqueous solution of either l-cysteine (to produce the final concentration of 120 μM) or GSH (to produce the final concentration of 10 mM). The resulting solutions were maintained in the dark. Afterward, aliquots of the final solutions were injected, using the autosampler of the LC–MS system with bypassed separation column, into the stream of 1:1 v/v methanol/water at 0.2 mL/min flow rate and immediately analyzed by ESI-MS in a positive ionization mode (mass range 100–1500 *m*/*z*). Samples from l-cysteine were analyzed on a Bruker amaZon SL mass spectrometer, while samples from GSH were analyzed on a Shimadzu LC–MS 8050 Liquid Chromatograph Mass Spectrometer. The obtained data were processed using the dedicated software provided by each respective manufacturer (Bruker, Data Analysis 4.4; Shimadzu, LabSolutions Connect software).

#### 3.2.4 Interaction with BSA (MALDI-TOF MS)

Bovine serum albumin (BSA, 30 μM in 50 mM NH_4_HCO_3_) was incubated with the selected complexes (**3a** or **3b**, 1 mM) at 37 °C for 48 h. Each complex was preliminarily dissolved in 50 μL of methanol. Following the incubation, an aliquot of the protein was adsorbed by repeated aspiration and release (20 times, 10-μL volume) into a ZipTip-C4 pipet tip (Merck, Darmstadt, Germany) that had been pre-equilibrated in 0.1% trifluoroacetic acid (TFA), washed by the equilibrating solution, and eluted into 10 μL of 70% acetonitrile containing 0.1% TFA. The eluate was subsequently subjected to evaporation using a vacuum centrifuge, and the residue was dissolved in 3 μL of 0.1% TFA. A 1 μL aliquot of this solution was deposited on an MSP Big Anchor 96 target plate (Bruker Daltonik, Bremen, Germany), onto which 1 μL of the matrix solution (sinapinic acid, 12 mg/mL in 0.1% TFA and 50% acetonitrile) was immediately superimposed. The sample was then allowed to dry. Mass spectra were acquired using a Microflex LRF20 MALDI-TOF mass spectrometer operated in a linear positive ion mode and controlled using flexControl 3.4 software from Bruker Daltonik). Protein Standard II, from Bruker Daltonik, was used for external calibration. All spectra were subjected to processing by using flexAnalysis 3.4 software from Bruker Daltonik.

### 3.3 Electrochemistry

Cyclic voltammetry measurements were conducted by using a PalmSens4 instrument connected to a computer operating with the PSTrace5 electrochemical software. Prior to use, anhydrous CH_2_Cl_2_ (Merck) was stored under argon over 3 Å molecular sieves. Both [N^*n*^Bu_4_]PF_6_ (electrochemical grade) and FeCp_2_, obtained from Fluka, were utilized without requiring additional purification. Cyclic voltammetries were carried out under argon by utilizing a 0.2 M solution of [N^*n*^Bu_4_]PF_6_ in CH_2_Cl_2_ as the supporting electrolyte. The working electrode and the counter electrode were comprised of a platinum disk and a platinum gauze, respectively, both enclosed within a glass tube. A leakage-free miniature electrode of Ag/AgCl/KCl (from eDAQ) was employed as a reference. The three-electrode designed cell was subjected to predrying via vacuum heating and then filled with argon gas. The Schlenk-type design of the cell was effective in upholding both anhydrous and anaerobic conditions. Subsequently, a solution of supporting electrolyte, prepared under argon, was introduced into the cell, and the CV of the solvent was acquired. Afterward, the analyte was introduced, and voltammograms were recorded. Under the selected experimental conditions, the one-electron oxidation of ferrocene occurred at a standard potential of *E*° = +0.45 V relative to Ag/AgCl/KCl.

### 3.4 Biological Studies

#### 3.4.1 In Vitro Cytotoxicity

The in vitro cytotoxicity of complexes (**2b**, **3a**, **3b**, **3c**, RAPTA-C, and cisplatin) was evaluated using the MTT assay with different incubation times. The cytotoxicity assessment was conducted on various human cancer cell lines, including A2780 ovarian carcinoma, A2780R cisplatin-resistant ovarian carcinoma, MCF-7 breast, HOS osteosarcoma, A549 epithelial lung, PANC-1 pancreatic, Caco-2 colorectal, PC-3 prostate adenocarcinoma, and HeLa cervical cell lines. Additionally, the assessment extended to a normal human fetal fibroblast cell line (MRC-5). Cells were obtained from ATCC collection and cultivated according to the producer’s instructions. Stock solutions of the compounds were prepared in DMF immediately before application, and diluted with H_2_O to achieve the final concentration of 0.1% v/v DMF.^99^ The culture media were enriched with 10% fetal calf serum. Subsequently, the cells were exposed to the tested compounds, vehicle (0.1% v/v DMF) and Triton X-100 (1% v/v), during incubation. The conventional MTT assay was carried out following different incubation periods (Tables 5-6). The absorbance was measured at 570 nm by using a spectrophotometer (Infinite M200, Schoeller Instruments, Prague, Czech Republic). The obtained data are presented as the percentage of cell viability with 100% representing negative control treatment (0.1% v/v DMF) and 0% representing the positive control (Triton X-100). The half-maximal inhibitory concentrations (IC_50_) were determined from dose–response curves using GraphPad Prism 6 software (GraphPad Software, San Diego, USA).

#### 3.4.2 Mechanism Studies

##### 3.4.2.1 Cell Culture

The A2780 ovarian carcinoma cell line was received from Merck (reference 93112519–1VL) and cultured at 37 °C in an environment with 5% CO_2_. The cultivation was carried out in complete cell culture medium (RPMI-1640 medium, Merck), which was supplemented with 10% fetal bovine serum (FBS, Merck), 1% l-glutamine (Merck) and 1% penicillin–streptomycin–neomycin (PSN, Merck). For the flow cytometry experiments, three independent duplicates were analyzed using a BD FACSVerse flow cytometer (Becton Dickinson, USA). At least 5 × 10^3^ events were recorded for each sample. The same procedure was replicated for the experiments described below. Solutions of the complexes were formulated using the stock solutions (Section 5.1). The reference sample for the drug was a solution of cisplatin at a concentration of 15 μM, whereas the negative control sample consisted of the vehicle alone (0.1% DMF).

##### 3.4.2.2 Cell Cycle Analysis

These studies were conducted on A2780 cells using the BD Cycletest Plus DNA kit (from Becton Dickinson, USA). Thus, cells were seeded in 96-well plates (10^4^ cells/well) and treated with 4 μM complexes **3a** and **3b** for 24 h. Following the incubation period, cells underwent a series of procedural steps. Initially, they were rinsed using PBS (0.1 M, pH 7.4). Subsequently, Solution A (comprising trypsin within a detergent buffer containing spermine tetrahydrochloride), Solution B (containing trypsin inhibitor and ribonuclease A within a citrate-stabilizing buffer with spermine tetrahydrochloride) and Solution C (comprising propidium iodide, PI, along with spermine tetrahydrochloride within a citrate stabilizing buffer) were sequentially introduced in accordance with the manufacturer’s instructions. A sample of cisplatin was employed as a positive control.

##### 3.4.2.3 Apoptosis and Caspase 3/7 Activation Analysis

Apoptosis assay was performed using an Annexin V-FITC/PI commercial kit (V13242, Thermo Fisher Scientific, USA), while caspase 3/7 activation assay was performed with a CellEvent Caspase-3/7 Green Flow Cytometry Assay Kit (C10427, Thermo Fisher Scientific, USA). A2780 cells were seeded in 24-well plates at a density of 5 × 10^4^ cells per well; after 24 h, the cells were exposed to 4 μM solution of the selected complex for 24 h. The drug-reference sample consisted of 15 μM solution of cisplatin, while vehicle (0.1% DMF) was employed as a negative control. The supernatant was collected, cells were washed with PBS (0.1 M, pH 7.4), and trypsin was added (0.25% trypsin-EDTA, GibcoTM). Finally, complete cell culture medium was added to stop the trypsinization, and a final volume of 500 μL was divided into two 250 μL aliquots, following the protocol provided by the manufacturer. Samples were centrifuged, treated with the respective buffers, and then incubated in the dark at room temperature for 10 min (Annexin V-FITC/PI) or at 37 °C for 30 min (CellEventTM Caspase-3/7 Green Detection Reagent). Heat-damaged cells (10 min, 60 °C) were used as a positive control for caspase 3/7 activation, while cisplatin was used as a positive control in the apoptosis assay.

##### 3.4.2.4 Autophagy Analysis

The potential induction of autophagy by **3a** and **3b** in A2780 cells was studied with the kit CYTO-ID Autophagy Detection 2.0 (from Enzo Life Sciences, USA), following the protocol provided by the manufacturer. Hence, a total of 5 × 10^4^ A2780 cells per well were seeded into a 24-well plate and after 24 h incubated with 4 μM solutions of the complexes, for 24 h. A 15 μM solution of cisplatin and vehicle (0.1% DMF) were employed as the positive and the negative control, respectively. The supernatant was collected, cells were washed with warm PBS, detached using 0.25% trypsin-EDTA (Gibco) and resuspended in a cell culture medium. Diluted CYTO-ID Green stain solution (according to the protocol) was used to stain the samples. Following incubation for 30 min in a light-protected environment, the samples were washed with PBS (phosphate-buffered saline). Subsequently, a flow cytometry experiment was conducted. A mixture of chloroquine (10 μM) and rapamycin (0.5 μM) was used as positive control.

##### 3.4.2.5 Mitochondrial Membrane Potential (MMP) Analysis

To investigate the effects on mitochondrial membrane potential of A2780 cells, an MITO-ID Membrane potential detection kit (Enzo Life Sciences, USA) was used. Cells were seeded in a 24-well (5 × 10^4^ cells/well), and then incubated with 4 μM solution of complexes for 24 h. The supernatant was collected, cells were washed with warm PBS, detached using 0.25% trypsin-EDTA (Gibco) and resuspended in a cell culture medium. Then, samples were stained with diluted MITO-ID MP Detection Reagent (according to the protocol) at RT for 15 min, prior to flow cytometry analysis. A positive control was established by treating the samples with 2 μM carbonyl cyanide 3-chlorophenylhydrazone.

##### 3.4.2.6 ROS production Assessment

ROS-ID Total ROS/Superoxide detection kit (from Enzo Life Sciences, US) was employed to quantify ROS and superoxide generation in A2780 cells following the treatment with the tested compounds. Thus, cells were seeded in a 96-well plate (10^4^ cells/well) and incubated with 4 μM solution of complexes for 24 h. A 100 μM solution of pyocyanin, which is a known oxidative-stress inducer, was used as a positive control. Following the treatment, the supernatant was aspirated, and the cells were subsequently rinsed with buffer (1X). The next step involved staining with the ROS/Superoxide Detection Solution, containing Oxidative Stress Detection Reagent (Green) and Superoxide Detection Reagent (Orange), both at 1:2500 dilution in wash buffer, at 37 °C in the dark for 60 min. Finally, a microplate reader Infinite M200Pro (Tecan, Switzerland) was used to measure sample fluorescence (green: Ex. 488 nm/Em. 520 nm, and red: Ex. 550 nm/Em. 610 nm) and three independent experiments were done in triplicates.

##### 3.4.2.7 Statistical Analysis

Three separate experiments were conducted, and the mean values along with the standard deviation (SD) were computed. Subsequently, statistical analysis was carried out using Statistica software^100^ involving One-way ANOVA followed by Dunnet post hoc test. Significant differences in comparison to the untreated control were denoted with asterisks (*p < 0.05, **p < 0.01, ***p < 0.001).

## Supporting Information Available

NMR spectra of products, UV–vis stability experiments, ESI-MS studies, and cyclic voltammograms (PDF)

## Notes

CCDC reference numbers 2259722 (**2b**) and 2259723 (**3a**) contain the supplementary crystallographic data related to the X-ray studies. This data is accessible free of charge at http://www.ccdc.cam.ac.uk/structures.
