# Supplementary material for: Anticancer Potential of Diruthenium Complexes with Bridging Hydrocarbyl Ligands from Bioactive Alkynols
Source: Inorg Chem. 2023 Sep 15;62(39):15875–90. doi: 10.1021/acs.inorgchem.3c01731 (PMC10548421; doi:10.1021/acs.inorgchem.3c01731)

Supporting Information

Anticancer Potential of Diruthenium Complexes with Bridging Hydrocarbyl Ligands from Bioactive Alkynols

Giulio Bresciani,[a] Ján Vančo,[b] Tiziana Funaioli,[a] Stefano Zacchini,[c] Tomáš Malina,[b] Guido Pampaloni,[a] Zdeněk Dvořák,[d] Zdeněk Trávníček,[b,*] Fabio Marchetti [a,*]

[a] University of Pisa, Dipartimento di Chimica e Chimica Industriale, Via G. Moruzzi 13, I-56124 Pisa, Italy

[b] Regional Centre of Advanced Technologies and Materials, Czech Advanced Technology and Research Institute, Palacký University, Šlechtitelů 27, CZ-779 00 Olomouc, Czech Republic

[c] University of Bologna, Dipartimento di Chimica Industriale "Toso Montanari", Viale del Risorgimento 4, I-40136 Bologna, Italy

[d] Department of Cell Biology and Genetics, Faculty of Science, Palacký University, Šlechtitelů 27, CZ-779 00 Olomouc, Czech Republic

*E-mail addresses: fabio.marchetti@unipi.it, zdenek.travnicek@upol.cz

Table of contents *Pages*

Figures S1-S9: NMR spectra of diruthenium complexes S2-S6

Figures S10-S19: UV-Vis experiments S7-S11

Figures S20-S26: Mass spectrometry studies S12-S18

Figure S27: Cyclic voltammetry profiles S19

Figure S1. ^{1}H NMR spectrum (401 MHz, acetone-d_6) of **2a**.

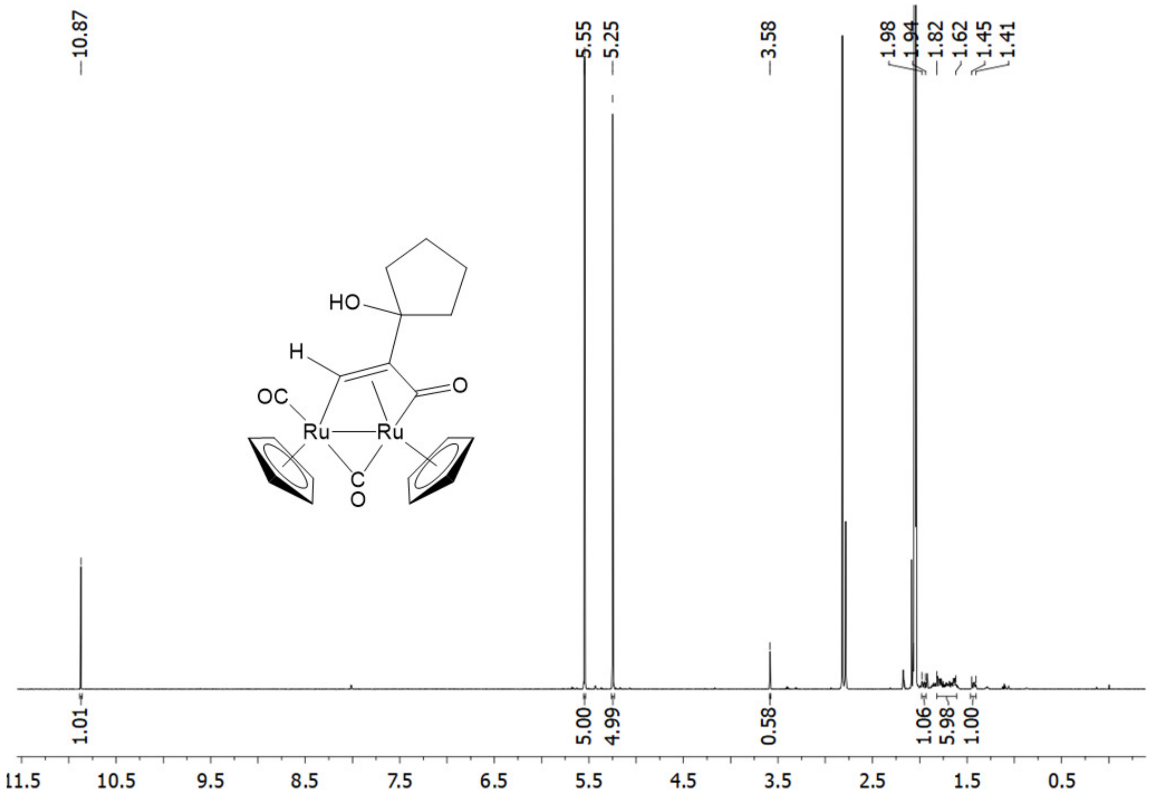

Figure S2. $^{13}C\{^{1}H\}$ NMR spectrum (101 MHz, acetone-d_6) of **2a**.

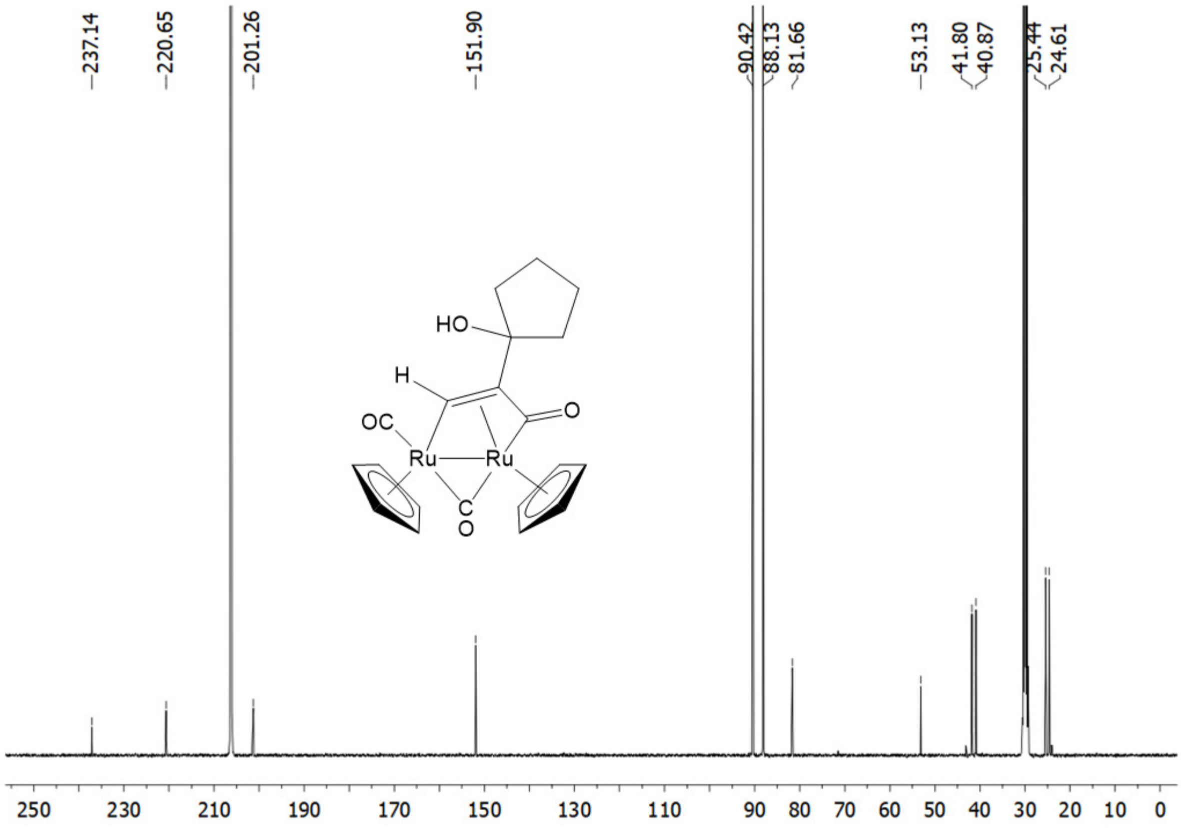

Figure S3. ^{1}H NMR spectrum (401 MHz, acetone-d$_6$) of **2b**.

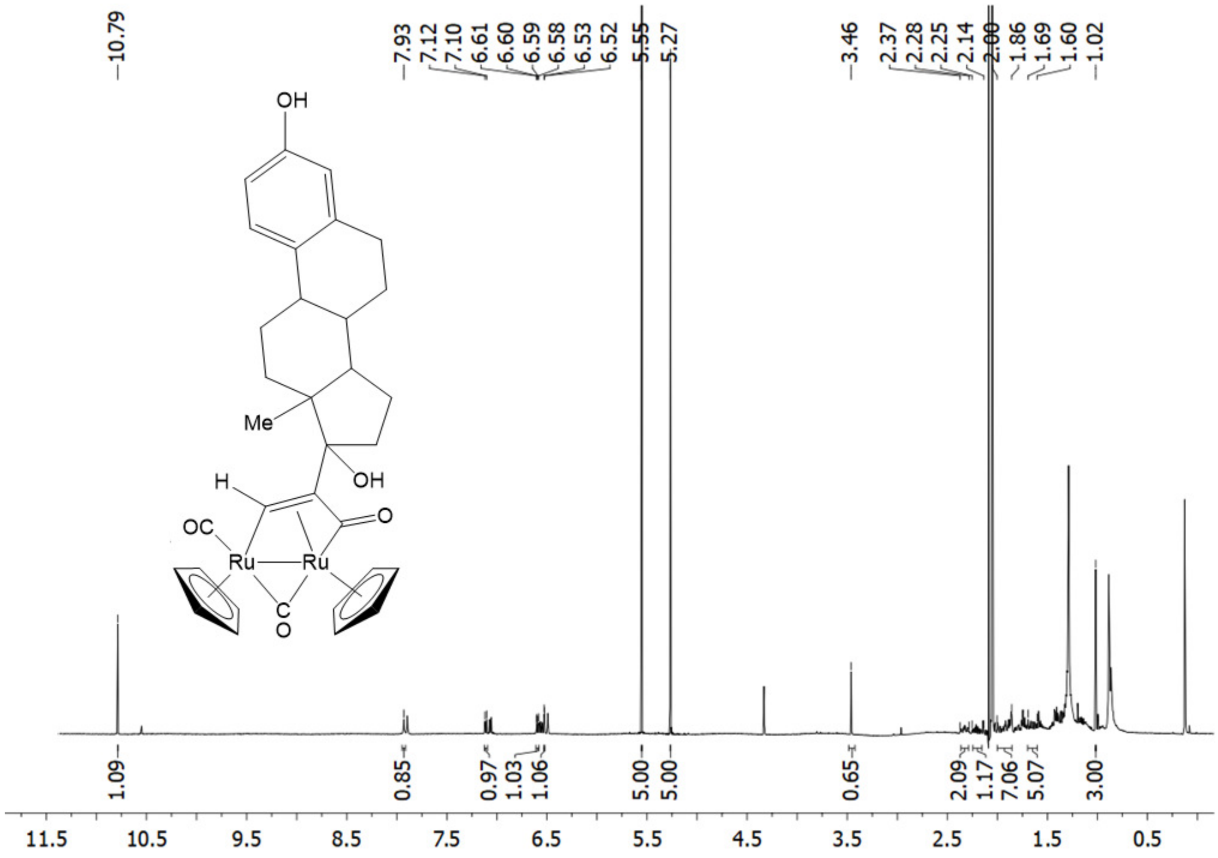

Figure S4. ^{13}C{^{1}H} NMR spectrum (101 MHz, acetone-d$_6$) of **2b**

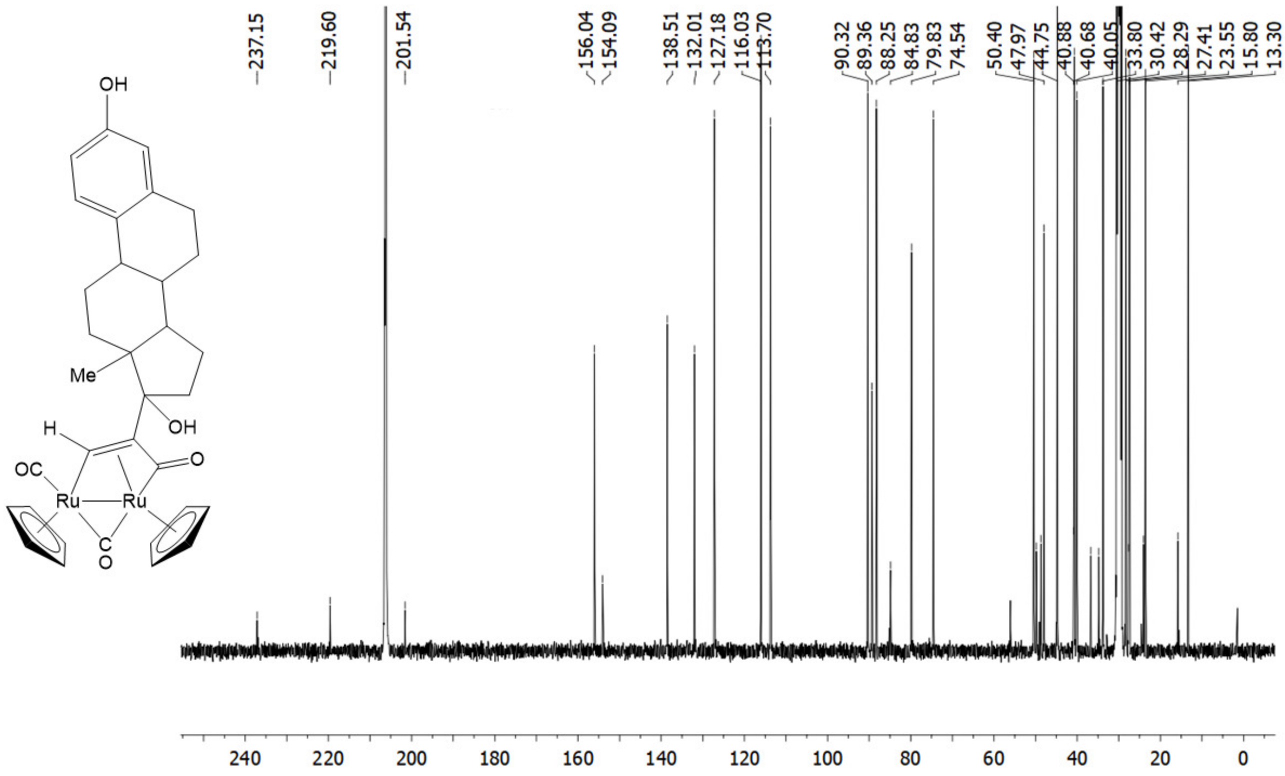

Figure S5. ^{1}H NMR spectrum (401 MHz, acetone-d$_6$) of **2c**.

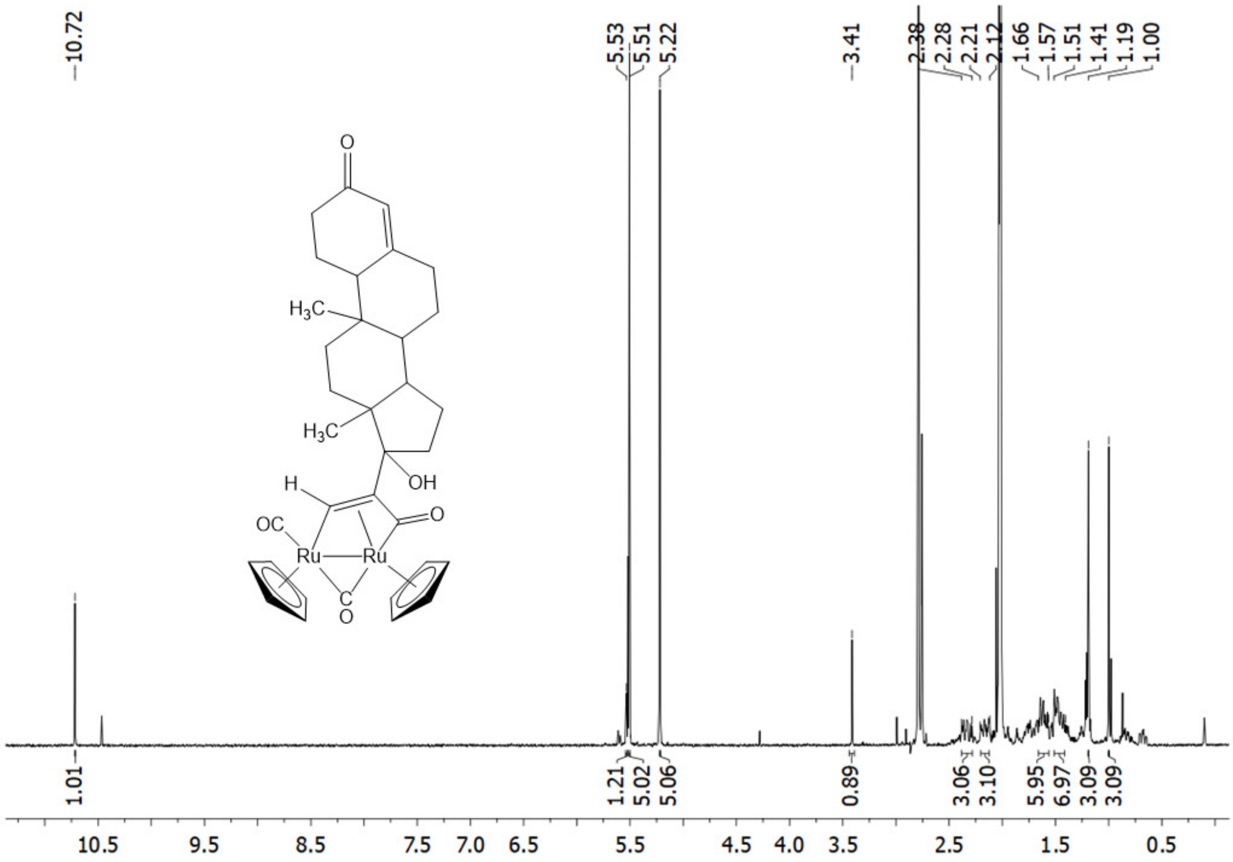

Figure S6. ^{13}C{^{1}H} NMR spectrum (101 MHz, acetone-d$_6$) of **2c**.

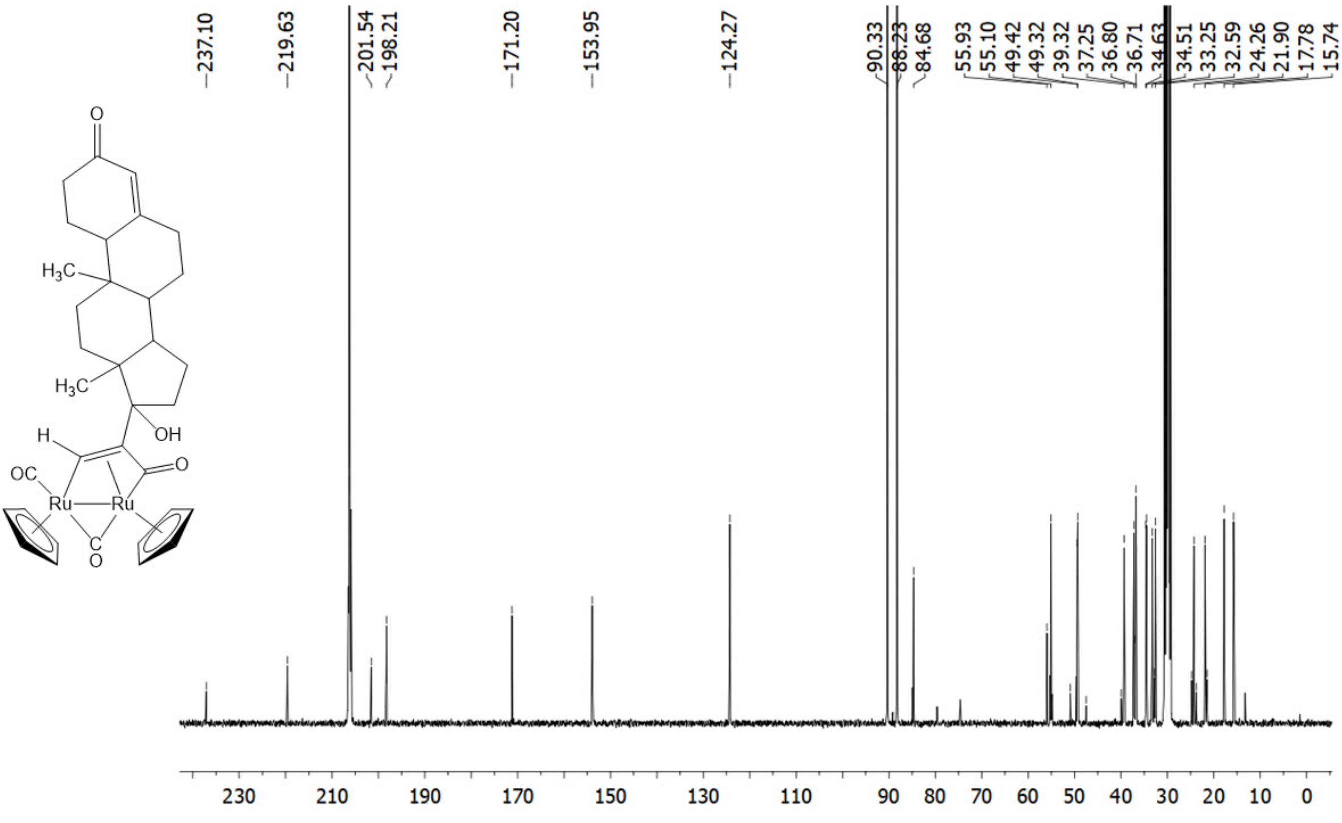

Figure S7. ^{1}H NMR spectrum (500 MHz, 253 K, acetone-d_6) of **3a**.

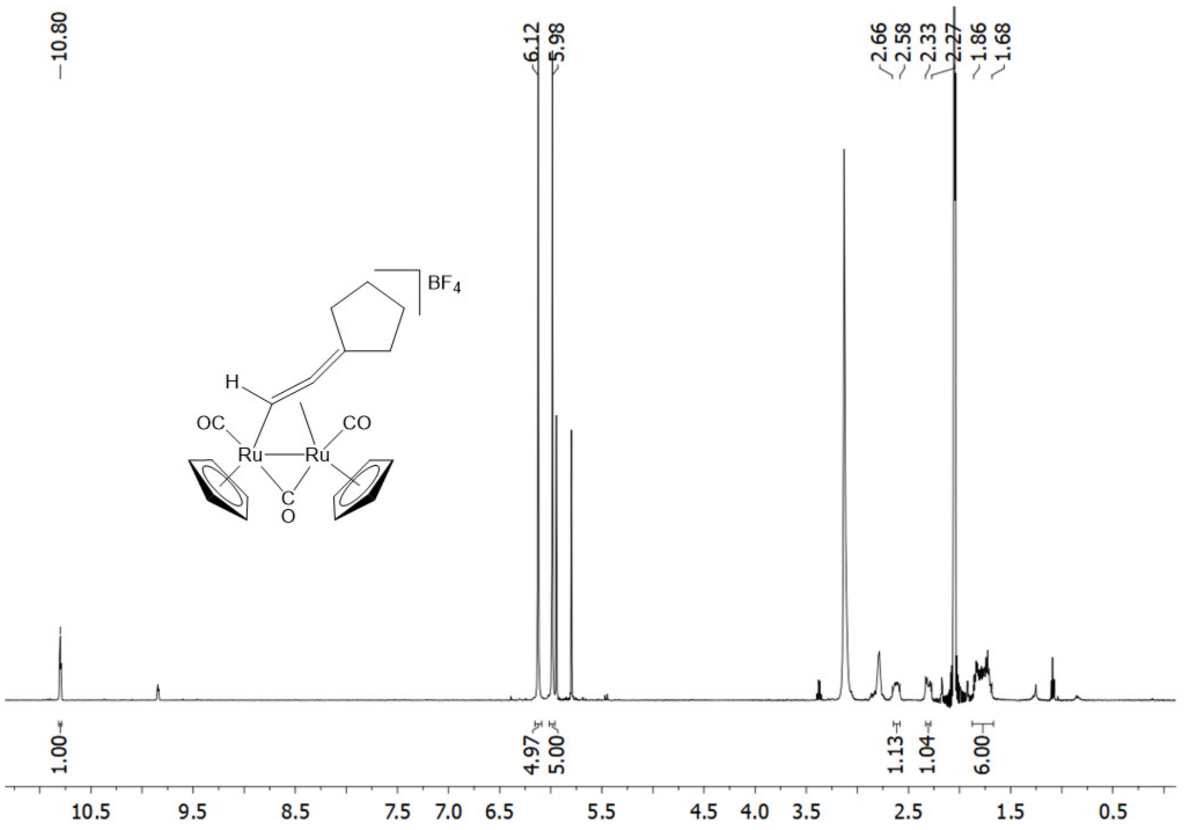

Figure S8. ^{1}H NMR spectrum (500 MHz, 253 K, acetone-d_6) of **3b**.

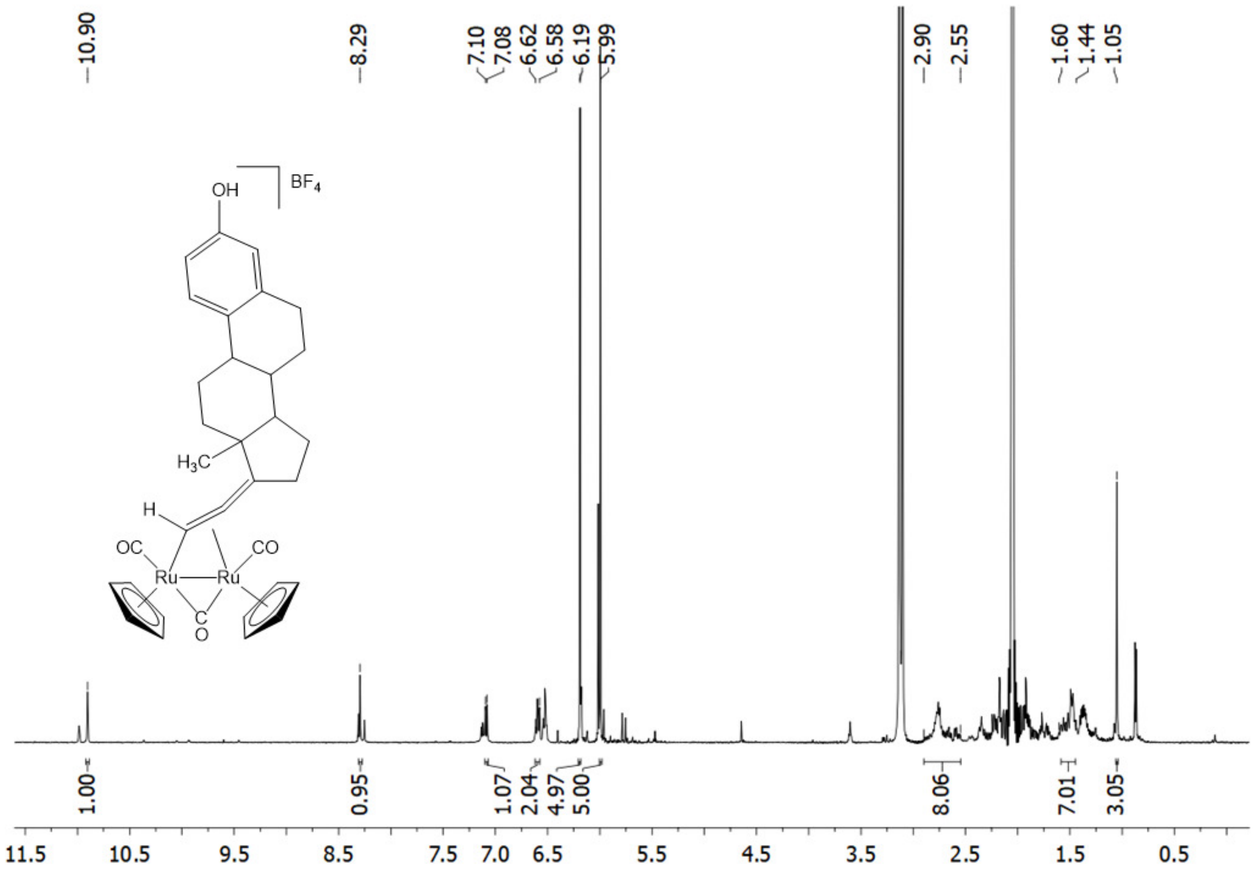

Figure S9. ^{1}H NMR spectrum (500 MHz, 253 K, acetone-d$_6$) of **3c**.

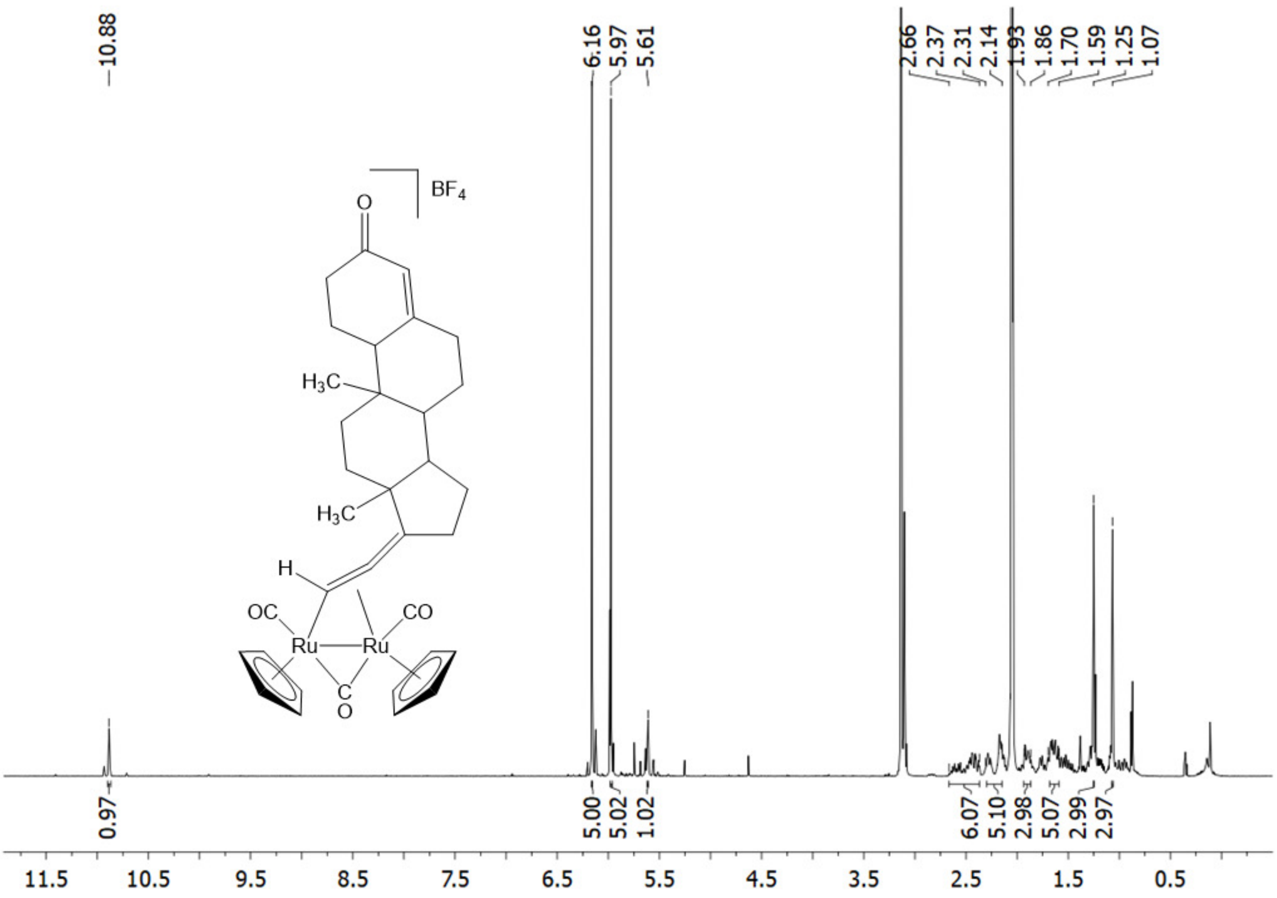

Figure S10. Comparison of UV-Vis spectra at different time of **2a** in **H_2O/CH_3OH** (5:1 v/v). Green line t = 0; blue line t = 30 minutes; black line t = 120 minutes; red line t = 18 hours

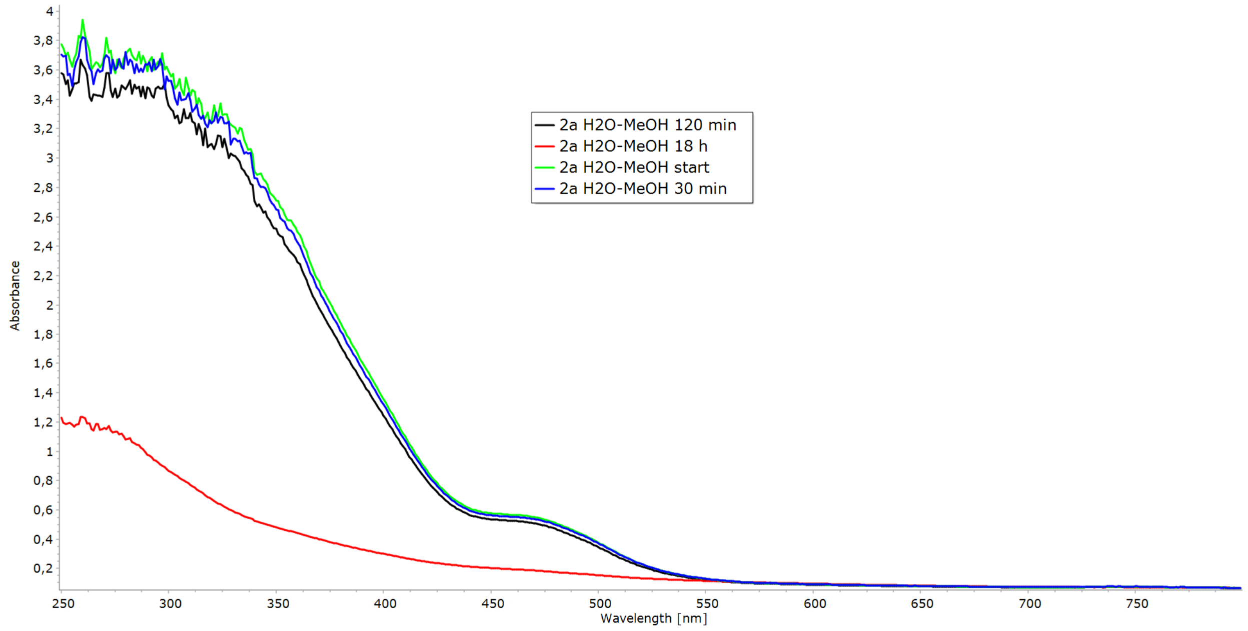

Figure S11. Comparison of UV-Vis spectra at different time of **2b** in **H_2O/CH_3OH** (5:1 v/v). Green line t = 0; black line t = 15 minutes; red line t = 30 minutes.

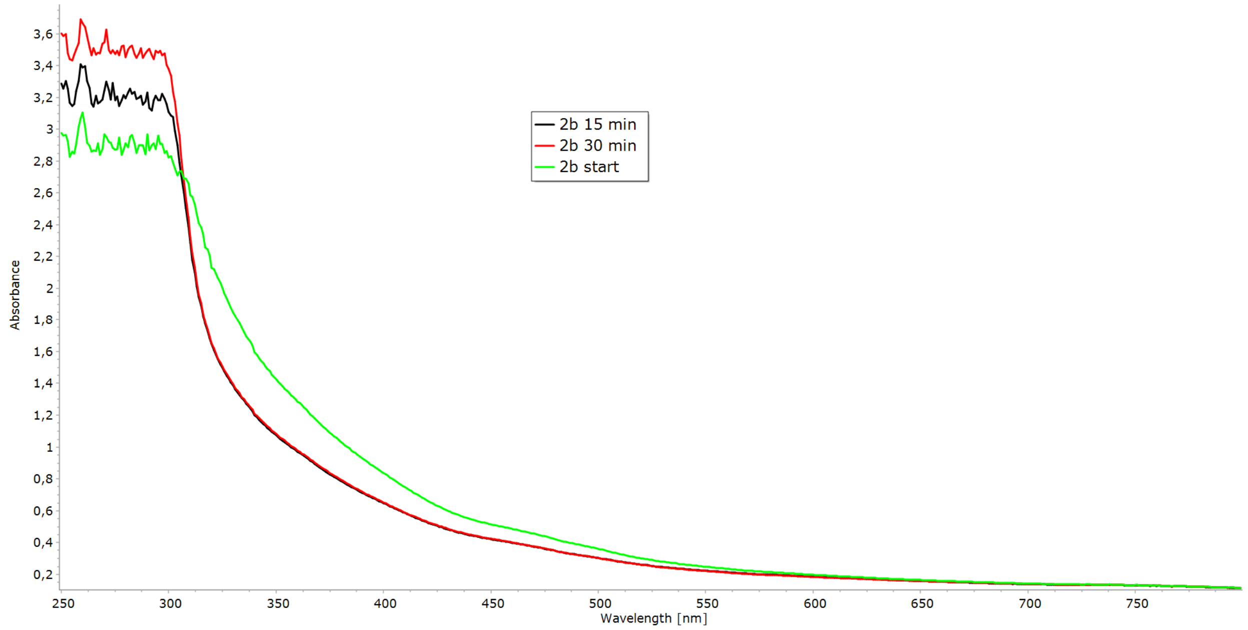

Figure S12. Comparison of UV-Vis spectra at different time of **2b** in **DMEM/CH_3OH** (5:1 v/v). Red line t = 0; black line t = 120 minutes; green line t = 18 hours.

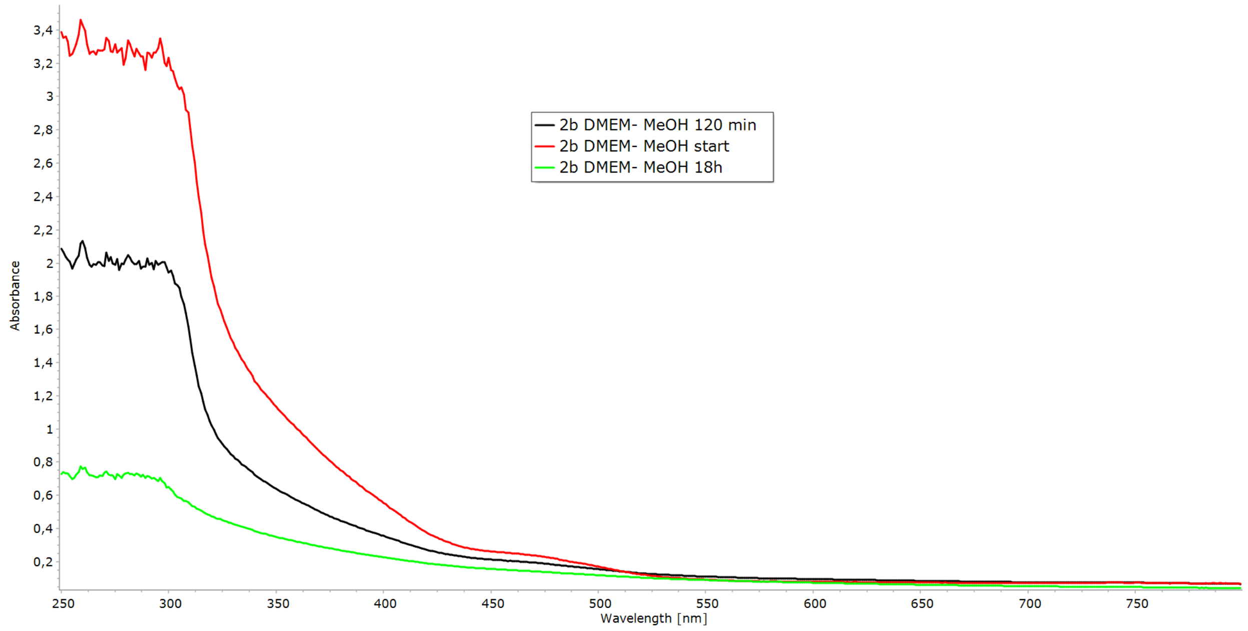

Figure S13. Comparison of UV-Vis spectra at different time of **2c** in **H_2O/CH_3OH** (5:1 v/v). Blue line t = 0; black line t = 30 minutes; green line t = 120 minutes; red line t = 18 hours

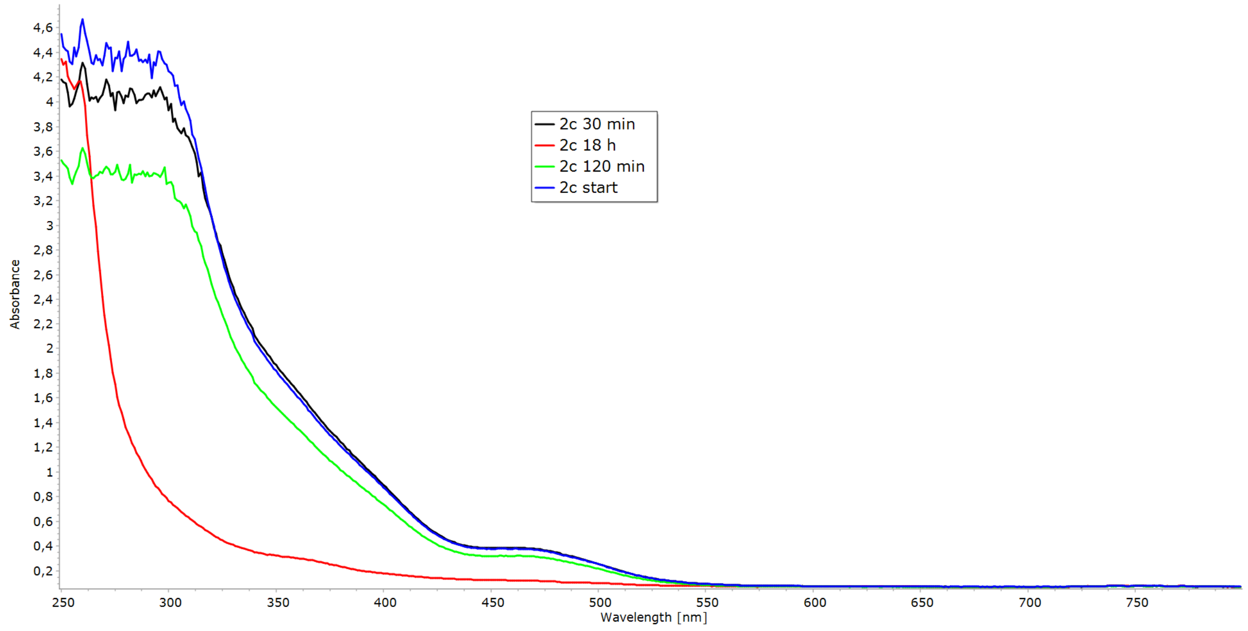

Figure S14. Comparison of UV-Vis spectra at different time of **3a** in **H_2O/CH_3OH** (5:1 v/v). Green line t = 0; black line t = 120 minutes; red line t = 18 hours

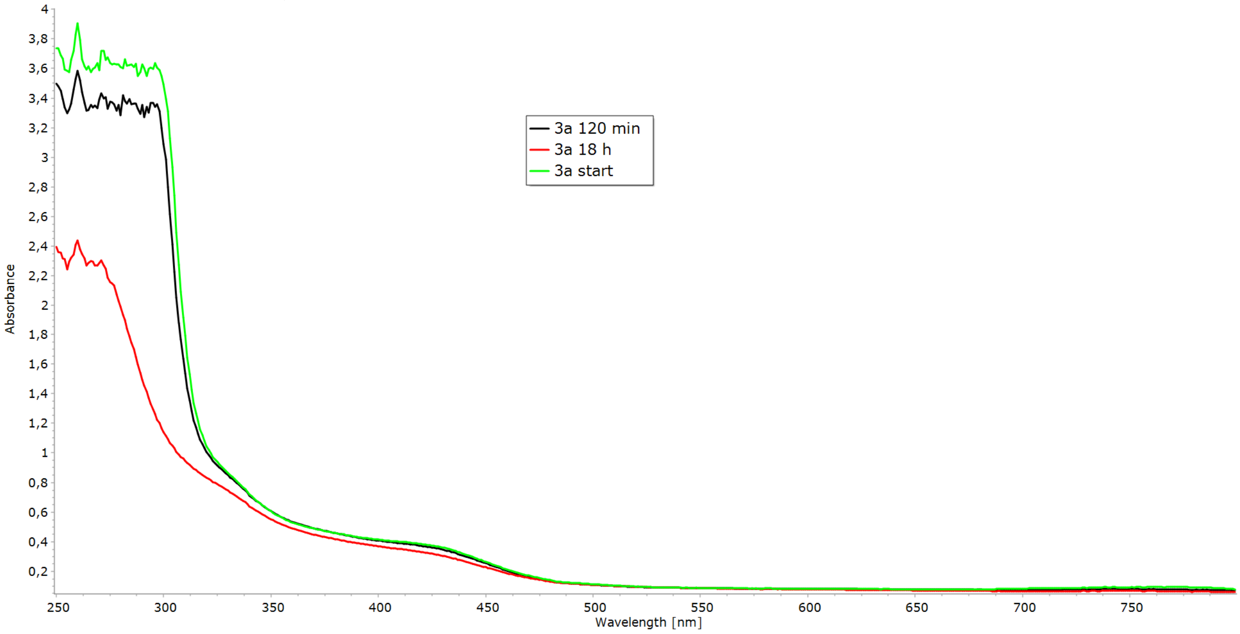

Figure S15. Comparison of UV-Vis spectra at different time of **3a** in **DMEM/CH_3OH** (5:1 v/v). Black line t = 0; red line t = 120 minutes; green line t = 18 hours.

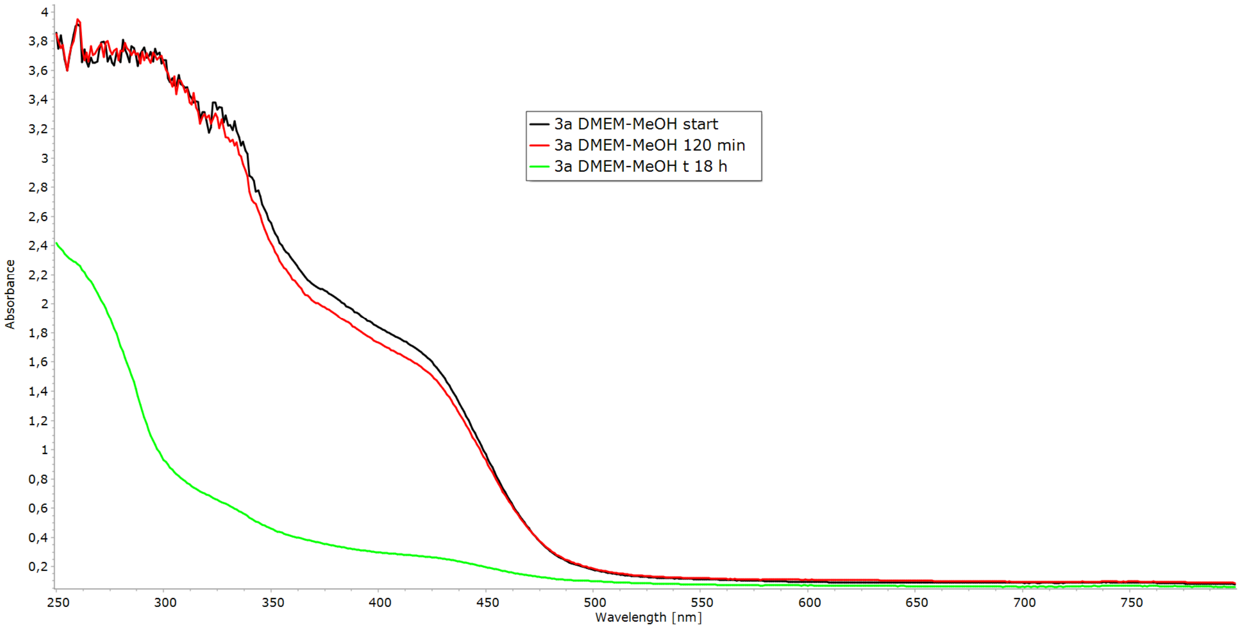

Figure S16. Comparison of UV-Vis spectra at different time of **3b** in **H_2O/CH_3OH** (5:1 v/v). Blue line t = 0; green line t = 30 minutes; black line t = 120 minutes; red line t = 18 hours;

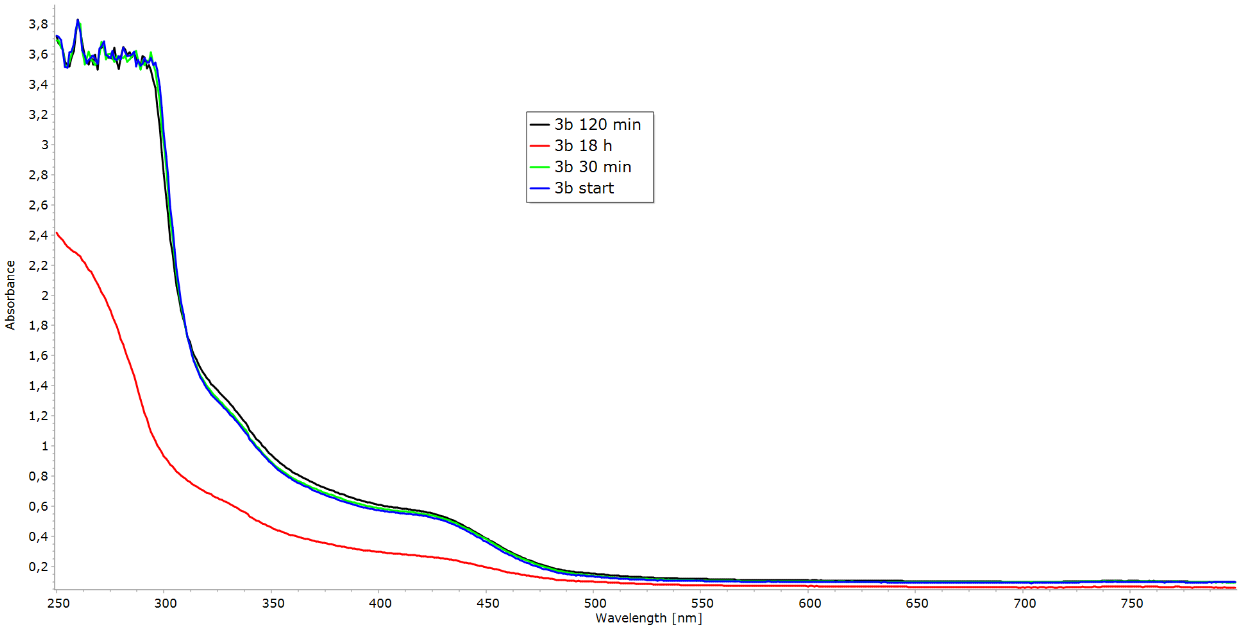

Figure S17. Comparison of UV-Vis spectra at different time of **3b** in **DMEM/CH_3OH** (5:1 v/v). Black line t = 0; red line t = 120 minutes; green line t = 18 hours

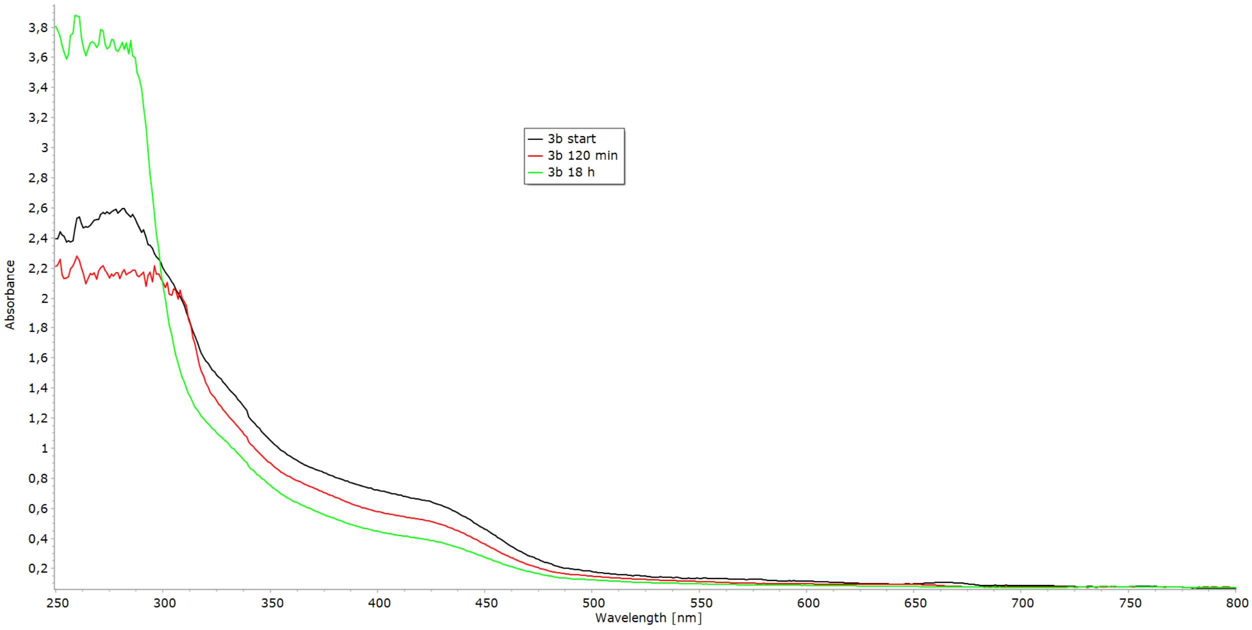

Figure S18. Comparison of UV-Vis spectra at different time of **3c** in **H_2O/CH_3OH** (5:1 v/v). Green line t = 0; black line t = 120 minutes; red line t = 18 hours

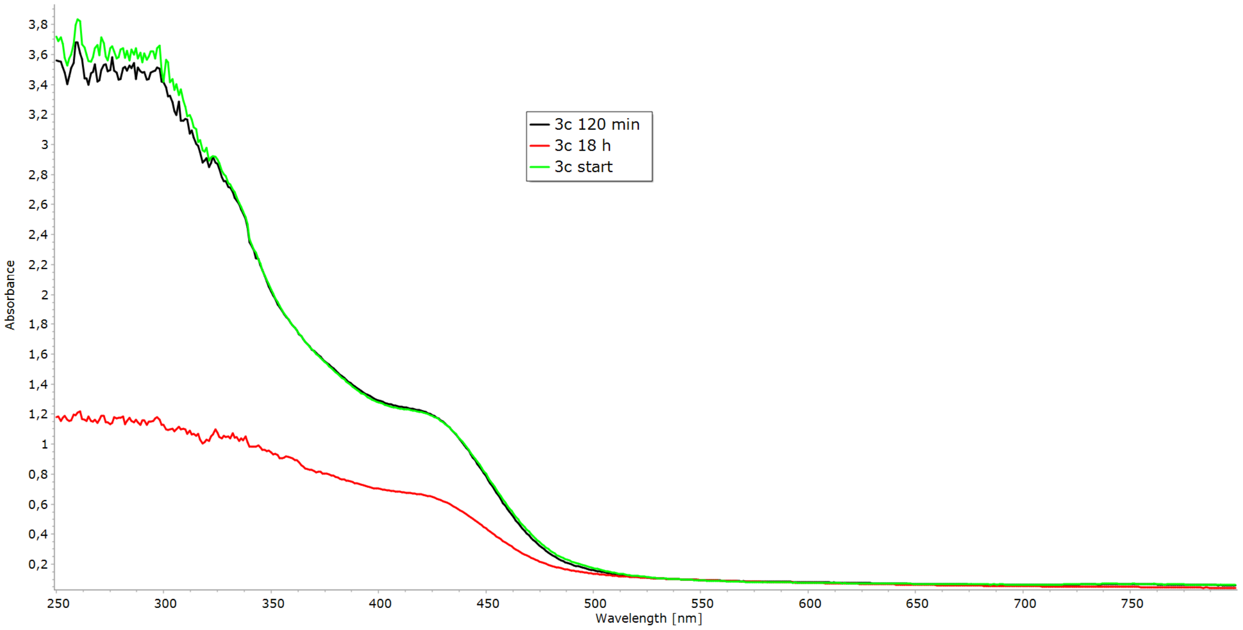

Figure S19. Comparison of UV-Vis spectra at different time of **3c** in **DMEM/CH_3OH** (5:1 v/v). Black line t = 0; red line t = 120 minutes; green line t = 18 hours

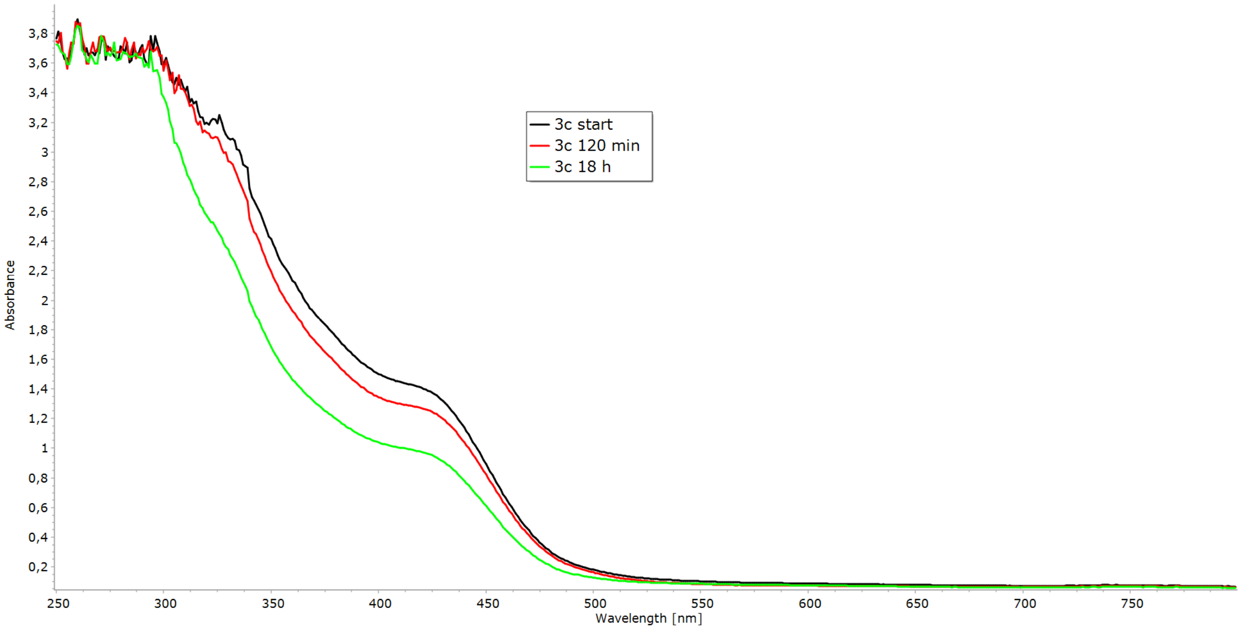

Figure S20. ESI-MS spectra of: **3a** in MeOH after 24h (panel A); **3a** in MeOH/H_2O (1:1 v/v) after 24h (panel B). The red-colored inset displays the theoretical isotopic distribution corresponding to the molecular peak of the cation of **3a**.

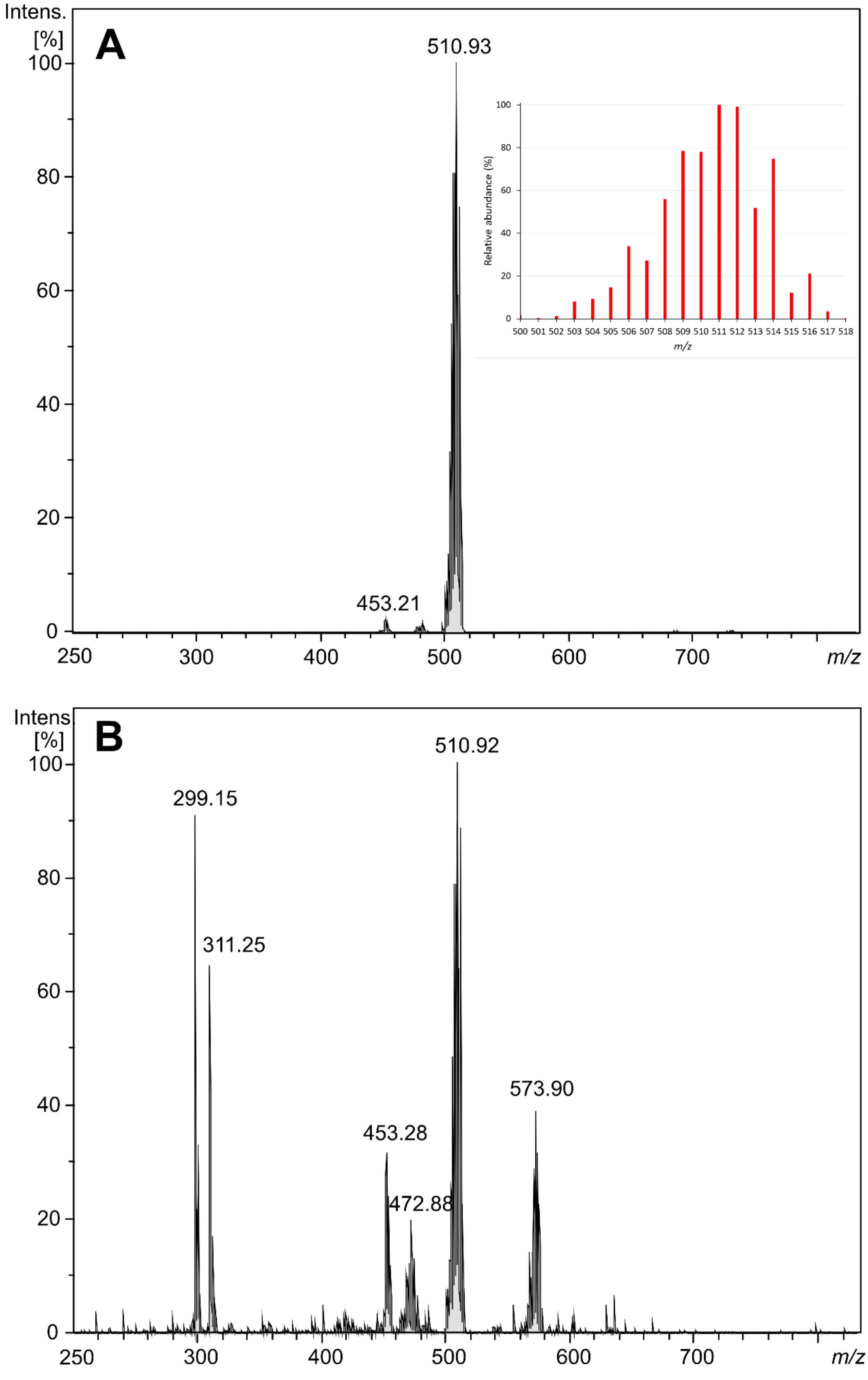

Figure S21. ESI-MS spectra of: **3b** in MeOH after 24h (panel A); **3b** in MeOH/H_2O (1:1 v/v) after 24h (panel B). The red-colored inset displays the theoretical isotopic distribution corresponding to the molecular peak of the cation of **3b**.

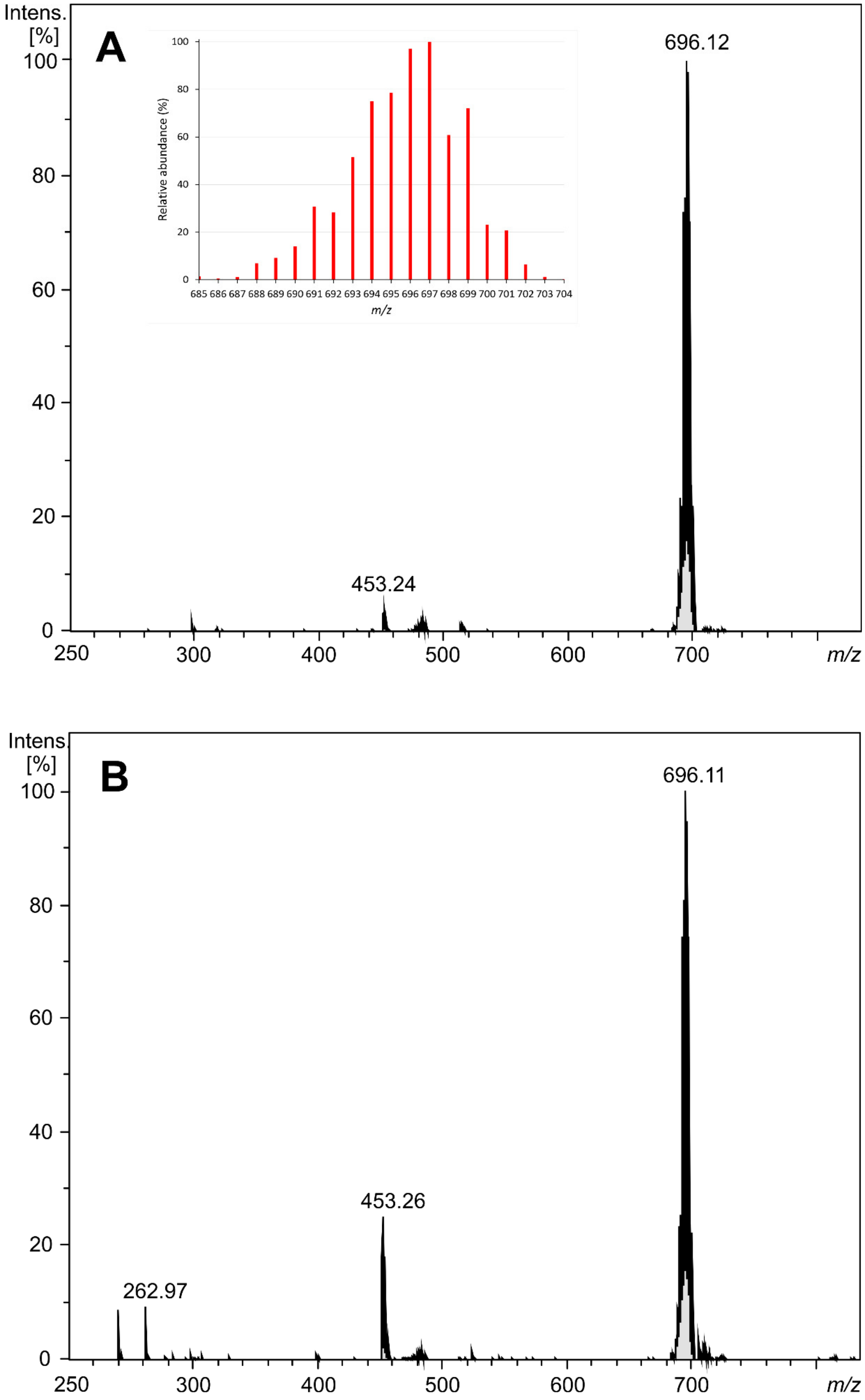

Figure S22. ESI-MS spectra: of **3c** in MeOH after 24h (panel A); **3c** in MeOH/H_2O (1:1 v/v) after 24h (panel B). The red-colored inset displays the theoretical isotopic distribution corresponding to the molecular peak of the cation of **3c**.

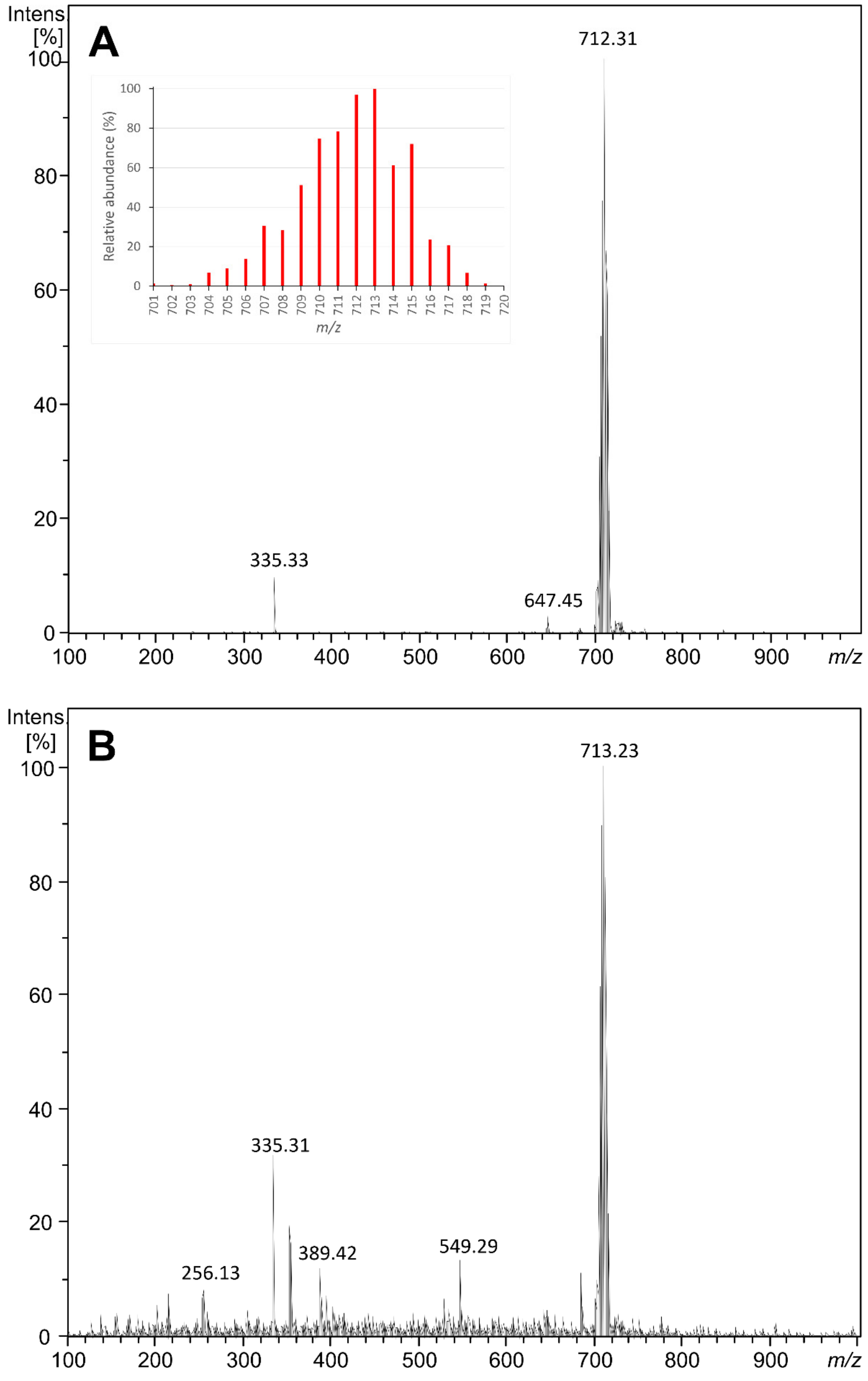

Figure S23. ESI-MS spectra of: **3a** in MeOH after 24h (panel A); **3a** (10 μM) after 2 h incubation with reduced glutathione (GSH, 10 mM) (panel B); **3a** (10 μM) after 24h incubation with GSH (10 mM) (panel C). The red color highlights the peak related to the cation of **3a**, while the green lines evidence the peaks related to GSH.

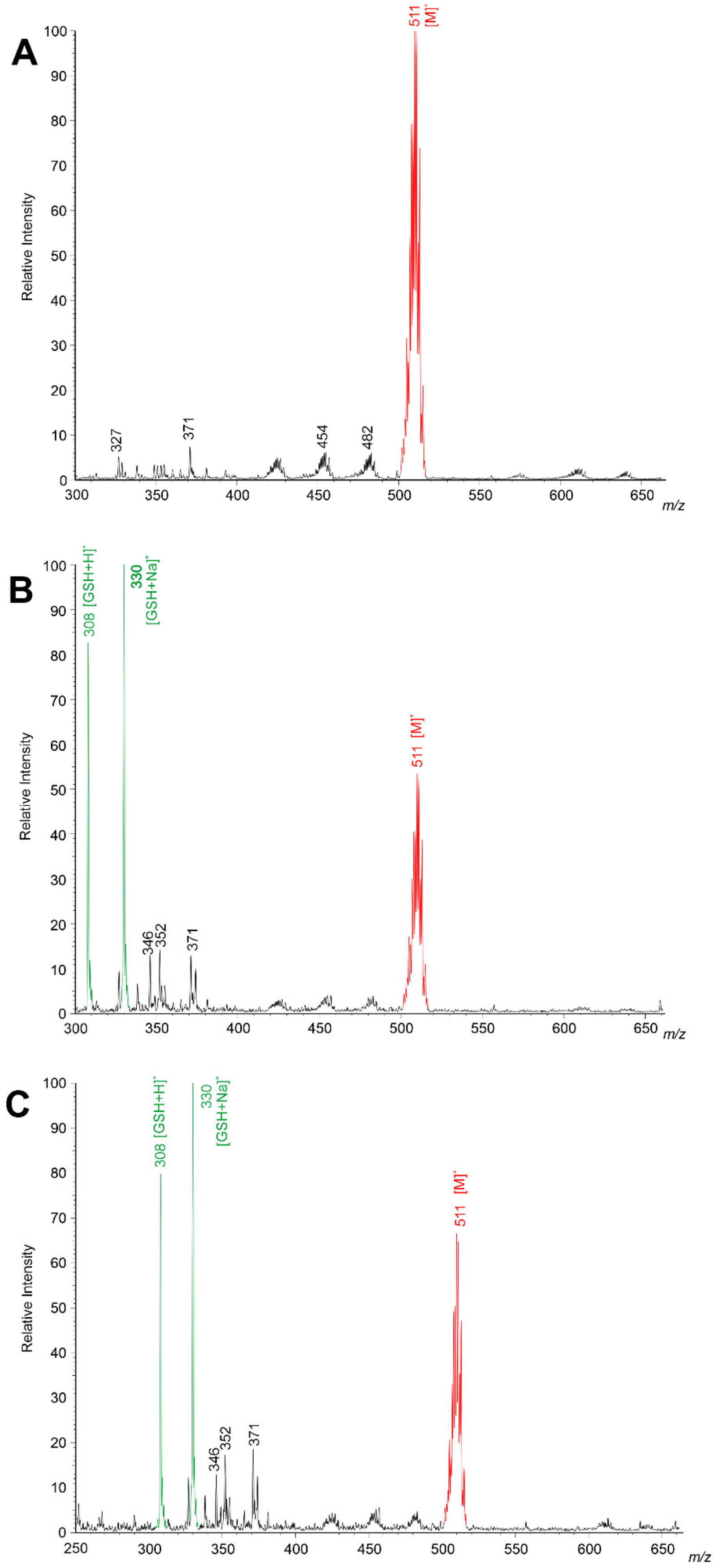

Figure S24. ESI-MS spectra of: **3b** in MeOH after 24h (panel A); **3b** (10 µM) after 2 h incubation with reduced glutathione (GSH, 10 mM) (panel B); **3b** (10 µM) after 24h incubation with GSH (10 mM) (panel C). The red color highlights the peak related to the cation of **3b**, while the green lines evidence the peaks related to GSH.

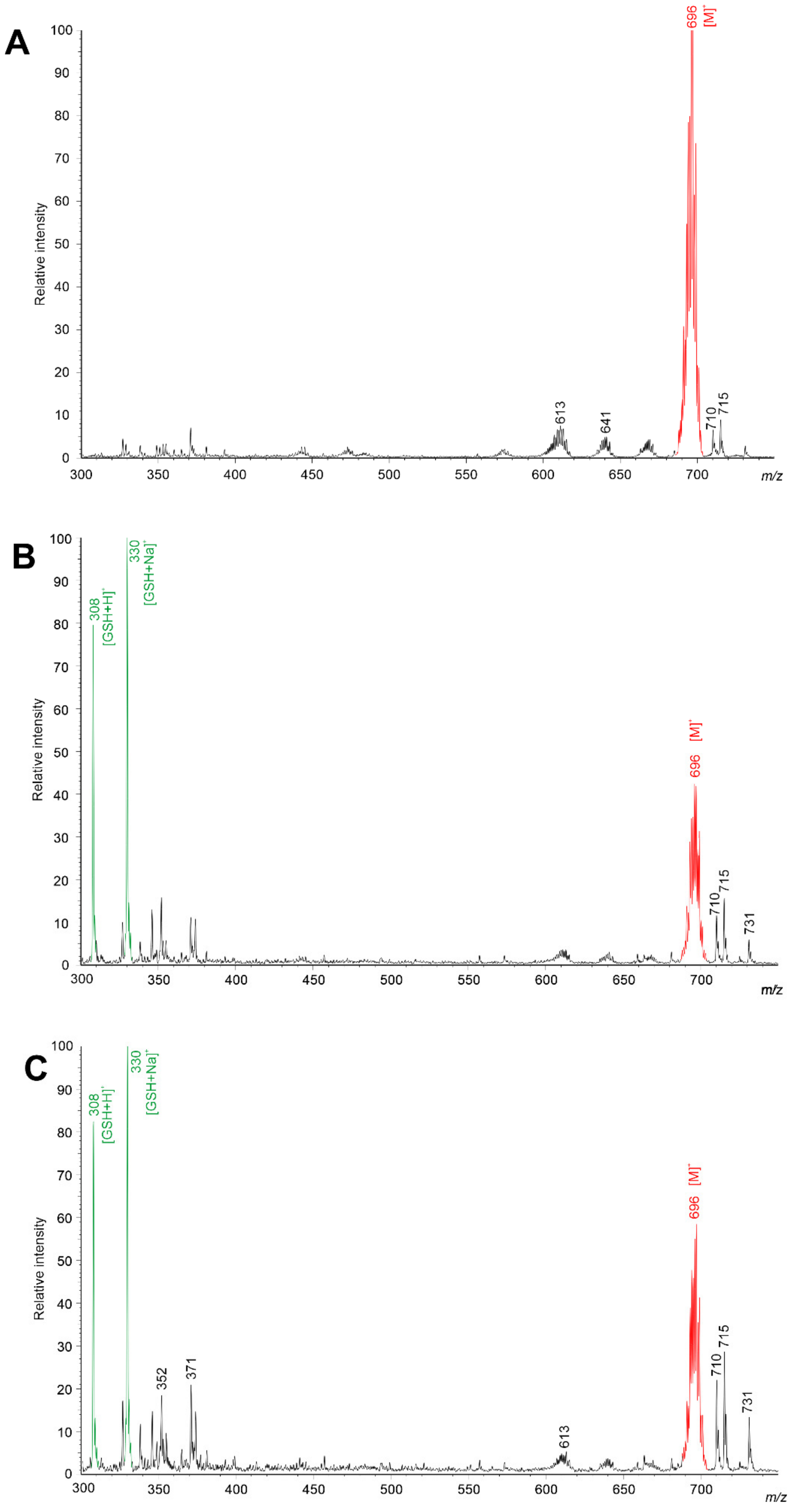

Figure S25. ESI-MS spectrum of MeOH/H_2O solution (1:1 v/v) of **3a** (10 μM) after 24h incubation with L-cysteine (120 μM) in the range 100-1000 *m/z* (panel A), and portion of the same spectrum in the range 400-600 *m/z* (panel B).

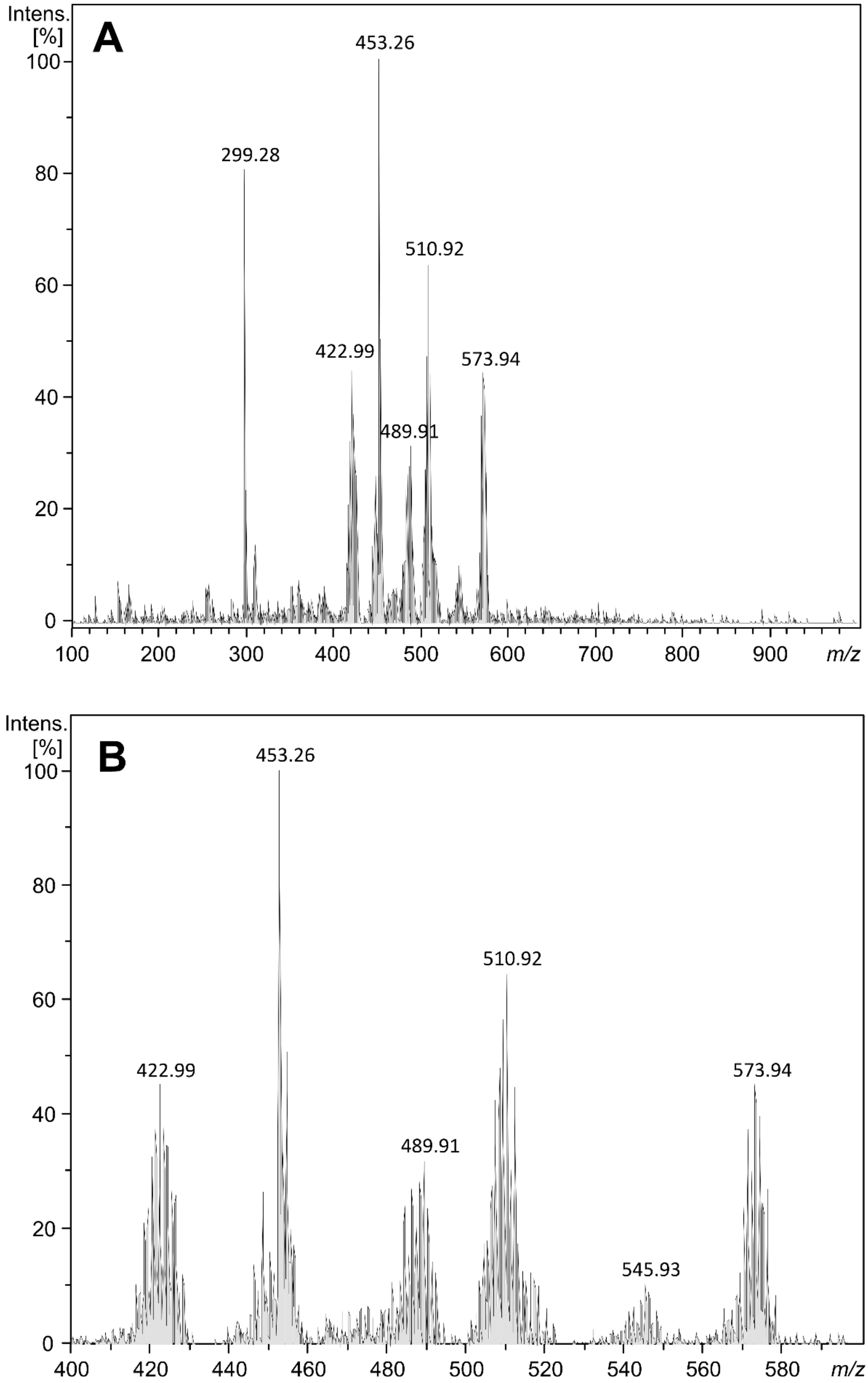

Figure S26. MALDI-TOF MS spectra of: native bovine serum albumin (BSA, *top panel*); **3b** after 24h incubation with BSA at 37 °C (*middle panel*); **3a** after 48h incubation with BSA at 37 °C (*bottom panel*). Each indicated value represents the average mass determined from six independent measurements ± standard deviation.

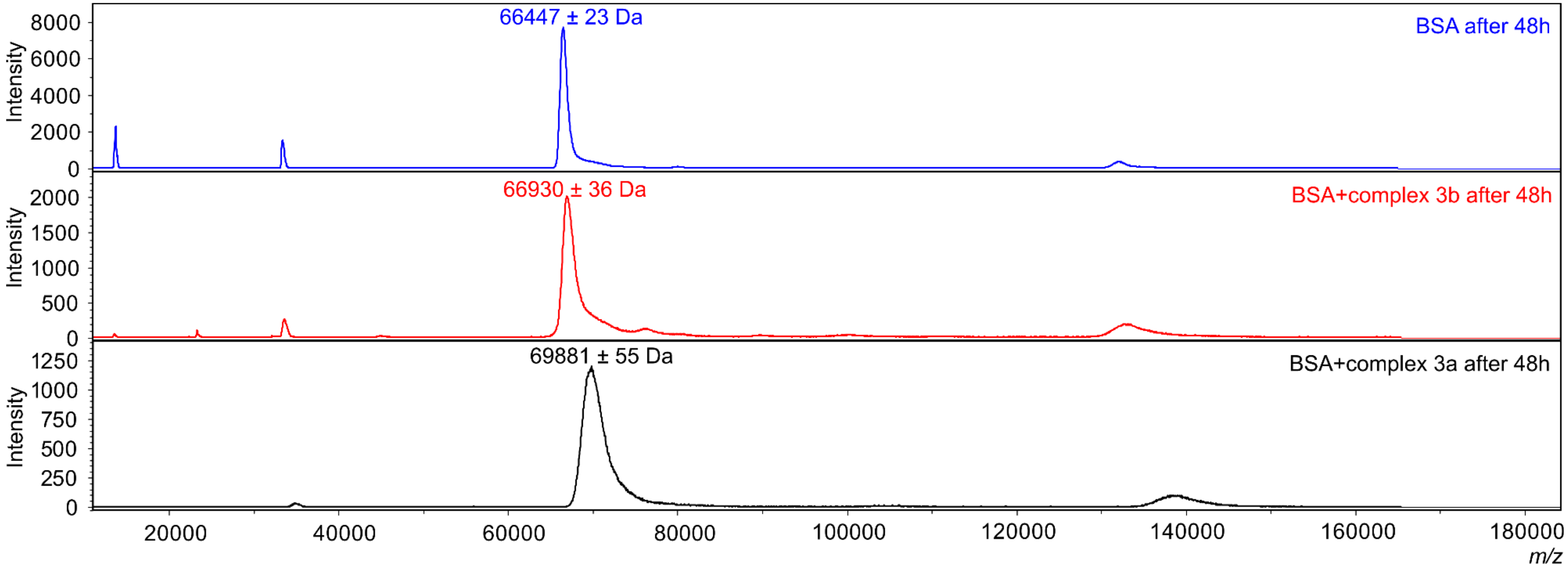

Figure S27. CV profiles recorded at a Pt electrode in CH_2Cl_2 solution of **2a** (blue line) and **3a** (red line). $[NBu_4][PF_6]$ (0.2 mol dm^{-3}) supporting electrolyte. Scan rate: 0.1 V s^{-1}.

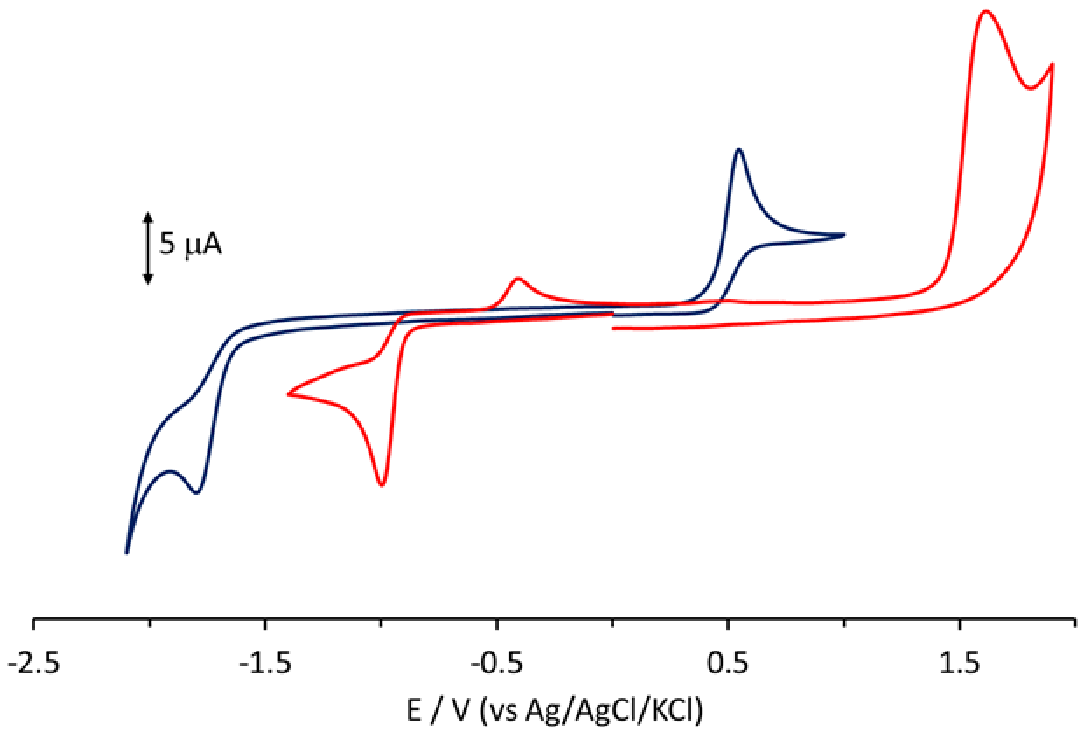

Supplement: Supplementary file 1 — ic3c01731_si_001.pdf [file ic3c01731_si_001.pdf]
